# Mutant p53^R270H^ drives altered metabolism and increased invasion in pancreatic ductal adenocarcinoma

**DOI:** 10.1172/jci.insight.97422

**Published:** 2018-01-25

**Authors:** Heather K. Schofield, Jörg Zeller, Carlos Espinoza, Christopher J. Halbrook, Annachiara del Vecchio, Brian Magnuson, Tania Fabo, Ayse Ece Cali Daylan, Ilya Kovalenko, Ho-Joon Lee, Wei Yan, Ying Feng, Saadia A. Karim, Daniel M. Kremer, Chandan Kumar-Sinha, Costas A. Lyssiotis, Mats Ljungman, Jennifer P. Morton, Stefanie Galbán, Eric R. Fearon, Marina Pasca di Magliano

**Affiliations:** 1 Department of Surgery,; 2 Program in Cellular and Molecular Biology,; 3 Medical Scientist Training Program,; 4 Department of Internal Medicine,; 5 Center for Molecular Imaging,; 6 Department of Radiology,; 7 Department of Molecular and Integrative Physiology, and; 8 Department of Biostatistics, School of Public Health, University of Michigan, Ann Arbor, Michigan, USA.; 9 Harvard University, Cambridge, Massachusetts, USA.; 10 Institute of Cancer Sciences, University of Glasgow, Glasgow, United Kingdom.; 11 Cancer Research UK Beatson Institute, Glasgow, United Kingdom.; 12 Department of Pathology,; 13 Comprehensive Cancer Center,; 14 Department of Radiation Oncology,; 15 Department of Environmental Health Sciences,; 16 Department of Human Genetics, and; 17 Department of Cell and Developmental Biology, University of Michigan, Ann Arbor, Michigan, USA.

**Keywords:** Gastroenterology, Oncology, Cancer, Mouse models, P53

## Abstract

Pancreatic cancer is characterized by nearly universal activating mutations in *KRAS*. Among other somatic mutations, *TP53* is mutated in more than 75% of human pancreatic tumors. Genetically engineered mice have proven instrumental in studies of the contribution of individual genes to carcinogenesis. Oncogenic Kras mutations occur early during pancreatic carcinogenesis and are considered an initiating event. In contrast, mutations in p53 occur later during tumor progression. In our model, we recapitulated the order of mutations of the human disease, with p53 mutation following expression of oncogenic Kras. Further, using an inducible and reversible expression allele for mutant p53, we inactivated its expression at different stages of carcinogenesis. Notably, the function of mutant p53 changes at different stages of carcinogenesis. Our work establishes a requirement for mutant p53 for the formation and maintenance of pancreatic cancer precursor lesions. In tumors, mutant p53 becomes dispensable for growth. However, it maintains the altered metabolism that characterizes pancreatic cancer and mediates its malignant potential. Further, mutant p53 promotes epithelial-mesenchymal transition (EMT) and cancer cell invasion. This work generates new mouse models that mimic human pancreatic cancer and expands our understanding of the role of p53 mutation, common in the majority of human malignancies.

## Abstract 2

This study shows that sequential mutations in Kras and Trp53 collaborate in pancreatic cancer and establishes effects of interrupting mutant Trp53 at different tumor stages.

## Introduction

Pancreatic cancer is a devastating disease and is the third leading cause of cancer-related death in the United States (1). Effective therapies are currently lacking, and the 5-year survival rate is less than 10%. Activating (oncogenic) mutations in *KRAS* are present in over 90% of human pancreatic cancers (2). *KRAS* mutations are present with high frequency in pancreatic intraepithelial neoplasias (PanINs), precursor lesions to pancreatic cancer, suggesting an initiating role for mutant KRAS (2, 3). In contrast, mutations in tumor suppressor genes such as *TP53* and *INK4A* are absent in low-grade PanINs and rare even in high-grade PanINs (3, 4). In invasive tumors *TP53* mutations are present in up to 75% of pancreatic cancers (5).

Cancer-associated missense mutations in the TP53 protein have pleiotropic contributions in tumorigenesis, including abrogation of the WT p53 protein’s ability regulate cell cycle checkpoints and apoptosis, and bypass oncogene-induced senescence (6). At the same time, these missense-mutant p53 proteins behave as gain-of-function mutants (for review, see ref. 7). In fact, different p53 mutant proteins have differing contributions to tumorigenesis in mouse lung cancer models (8). In pancreatic cancer, missense mutations are most common, although null alleles are occasionally observed (5, 9).

Mice expressing a conditionally activated, oncogenic Kras^G12D^ protein in pancreatic epithelial cells (Ptf1a-Cre;LSL-Kras^G12D^, known as KC mice) develop PanINs — similar to their human counterparts — with incomplete penetrance and long latency to malignancy (10). Simultaneous expression of mutant Kras^G12D^ expression and mutant p53^R172H^ — in the model known as KPC — results in the development of metastatic pancreatic cancer with high penetrance and a latency of only a few months (11) as mutant p53 bypasses oncogene-induced senescence (12). Alternative approaches to the combination of mutant Kras and p53 use inducible Cre systems that allow expression of the oncogenes in the adult pancreas (13, 14). Common to all of these models is that the mutations in *Kras* and *Tp53* are introduced at the same time. Here, we describe a model where we mimic the sequence of mutations observed in the human disease, by introducing the *Tp53^R270H^* mutation in PanIN-bearing animals. Further, by using a reversible, doxycycline-regulated (dox-regulated) expression system, we inactivated p53^R270H^ expression at different stages of carcinogenesis. This system allowed us to investigate the effect of sequential introduction of mutations in pancreatic cancer, as well as study the role of mutant p53 at different stages of carcinogenesis.

Importantly, in our model we have chosen to introduce the *Tp53*^R270H^ mutation, ortholog to human R273H, as position 273 is the most commonly mutated amino acid in pancreatic cancer, as well as in other common human tumors, such as colon adenocarcinoma (15).

## Results

### Mutant p53^R270H^ synergizes with oncogenic Kras to promote pancreatic cancer progression.

**Figure 1:**
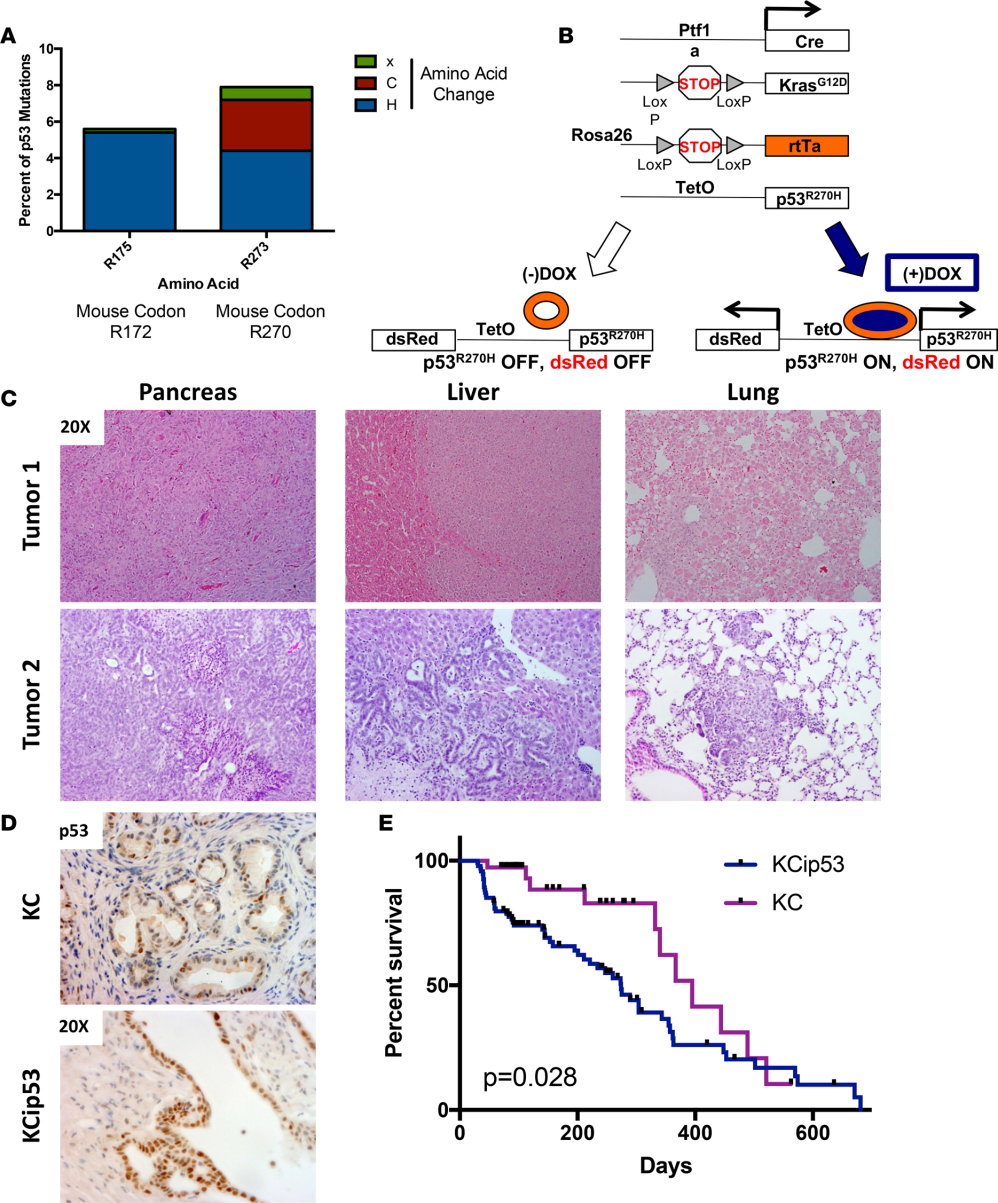
KCip53 mice recapitulate the stages of human pancreatic cancer. (**A**) Percentage of human pancreatic tumor samples with p53 R175 or R273 mutations, from the COSMIC database. (**B**) Scheme of Ptf1a-Cre;LSLKras^G12D^;TREp53^R270H^;R26^rtTa/rtTa^ animals, termed KCip53 here. (**C**) H&E from pancreas, liver, and lung from two separate KCip53 animals with tumors. (**D**) IHC for p53 in KC and KCip53 tissue. (**E**) Survival curve for KC and KCip53 animals. *n* = 94 mice for KCip53, *n* = 37 for KC. Survival significance analysis by log-rank test. All original magnification for histology at ×20, as shown in images. TetO, tetracycline operator.

We classified the spectrum of TP53 mutations in human pancreatic cancer using publicly available data in the Catalogue of Somatic Mutations in Cancer (COSMIC) database (http://cancer.sanger.ac.uk/cosmic). As in many other human malignancies, the majority of *TP53* mutations in pancreatic cancer are missense substitutions in the DNA-binding domain of the p53 protein (5). The most common Tp53 missense mutation in this data set was p53^R175H^, which corresponds to mouse p53^R172H^. However, the most frequently mutated codon in pancreatic cancer was codon 273. Substitution of histidine for arginine (R273H) is the most common substitution at that position. The corresponding change in the mouse gene is R270H (Figure 1A).

To explore the role of p53^R270H^ in pancreatic cancer, we designed a construct to express this mutant, as well as the fluorescent reporter dsRed, under the control of a tetracycline-responsive element (TRE). We initially verified the functionality of the TREp53^R270H^ cassette in tissue culture. HEK-293 cells were transfected with individual plasmids encoding for the reverse tetracycline transactivator (rtTa) and the TREp53^R270H^ construct. Accumulation of p53 protein was observed by immunoblot in dox-treated cells (Supplemental Figure 1A; supplemental material available online with this article; https://doi.org/10.1172/jci.insight.97422DS1). We then generated transgenic mice carrying the TREp53^R270H^ expression construct. Transgenic TREp53^R270H^ animals were crossed with the Krt5-tTa mouse strain, which expresses tTa in the skin, to establish in vivo regulation of the transgene. Expression of both p53 and dsRed was observed by IHC in the Krt5-tTa; TREp53^R270H^ animals and not in control littermates (Supplemental Figure 1B). Therefore, the TREp53^R270H^ construct allows for mutant p53 expression in an inducible manner in vitro and in vivo.

To assess the effects of expressing mutant p53^R270H^ in the context of mutant Kras pancreata, we generated Ptf1a-Cre;LSL-Kras^G12D^;TREp53^R270H^;R26^rtTa/rtTa^ mice, herein designated as KCip53 mice. The Ptf1a-Cre allele expresses Cre recombinase in the pancreatic epithelium, thus leading to expression of both Kras^G12D^ and rtTa. Administration of dox to the mice induces rtTa-mediated transcriptional activation of p53^R270H^. A dsRed reporter is expressed at the same time as mutant p53, allowing for a surrogate marker of dox-inducible gene expression (Figure 1B). KCip53 animals were given water or chow containing dox at approximately 4 weeks of age, and the animals were maintained on continuous dox administration. KC littermates were similarly kept on dox for the duration of the experiments, to control for other potential effects of the antibiotic. Starting at 8 months of age, KCip53 animals developed large pancreatic tumors with metastases to the liver and lung (Figure 1C). Accumulation of p53 protein in the tumors was confirmed by IHC analysis comparing PanIN lesions in KCip53 mice with lesions in KC mice (Figure 1D). In KC mouse lesions, p53 protein accumulation was occasionally present, as previously observed, likely indicating the accumulation of p53 in response to oncogenic stress induced by Kras^G12D^ (6). In KCip53 compared with KC lesions we observed increased accumulation of p53, although intriguingly only in a subset of cells, similar to the Krt5-tTa; TREp53^R270H^ animals (Figure 1D and Supplemental Figure 1B). To determine whether the expression of mutant p53 in this model affected tumor progression, we aged the animals until they reached humane endpoints. KCip53 mice had a median survival of 275 days, compared to 395 days in KC mice (Figure 1E, *n* = 94 mice for KCip53, *n* = 37 for KC). Notably, the time to tumor development in KCip53 mice was variable, as was the metastatic load and location. Of the animals that were evaluated histopathologically, liver metastases were observed in 8 of 22 animals and lung metastasis in 6 of 19 (see table in Supplemental Figure 2). Thus, expression of p53^R270H^ accelerated Kras-induced tumorigenesis and led to the development of metastatic disease.

The TREp53^R270H^ allele in the KCip53 animals is a transgene, and therefore the animals initially retain two WT alleles of *Trp53*, potentially mitigating some of the effects of mutant p53^R270H^ expression. To mimic the human scenario, where one allele of *TP53* is mutated and only one WT allele is present, we generated animals with the genotype Pdx1-Cre; LSL-Kras^G12D^; LSL-Trp53^R270H^, called KP^R270H^C here (Supplemental Figure 3A). In brief, the sequence encoding for p53^R270H^ was inserted in the p53 locus, preceded by a floxed stop cassette. Thus, the mutant p53 was expressed upon Cre recombination in the pancreas at the same time as mutant Kras, in a model similar to the KPC model (11). KP^R270H^C mice, similar to KCip53 animals, have a shortened life span compared with KC littermates (Supplemental Figure 3B, *n* = 43 KP^R270H^C and *n* = 39 KC). Histology from mice at necropsy revealed extensive tumor burden in the KP^R270H^C animals, with metastatic lesions in the liver and lungs (Supplemental Figure 3C). Thus, analysis of this model corroborates the phenotype of the KCip53 mouse.

As an alternative approach, we generated KCip53 animals with one null copy of p53. The genotype of these mice is Ptf1a-Cre;LSLKras^G12D^;TREp53^R270H^;R26^rtTa/rtTa^;p53^fl/+^, and we refer to them herein as KCip53;p53^fl/+^. Given the complexity of the breeding scheme, we obtained only a relatively small cohort of these mice (*n* = 18). We observed a trend toward shorter survival in these mice compared with KCip53 mice, although the difference was not statistically significant (Supplemental Figure 4A). In 4 of 4 KCip53;p53^fl/+^ mice that were evaluated histopathologically, we did not observe evidence of metastasis (Supplemental Figure 4B). However, these animals had large, locally invasive primary tumors (Supplemental Figure 4C). The data thus support the notion that the number of WT copies of p53 present delays the onset of tumorigenesis.

### Mutant p53^R270H^ promotes formation, progression, and maintenance of PanINs.

Given that mice expressing mutant p53^R270H^ developed cancer earlier than KC littermates, we sought to determine whether this effect was associated with earlier PanIN development. To verify the mechanics of the KCip53 system, we analyzed a small cohort of animals that had never been on dox, and therefore should never have had mutant p53^R270H^ expression. KCip53 animals were analyzed at 10 weeks of age. The resulting histology was similar to that of KC mice, suggesting that, as would be expected, KCip53 animals not on dox did not have expression of mutant p53^R270H^ (Supplemental Figure 5, *n* = 3 KC on dox, *n* = 4 KCip53 never on dox).

**Figure 2:**
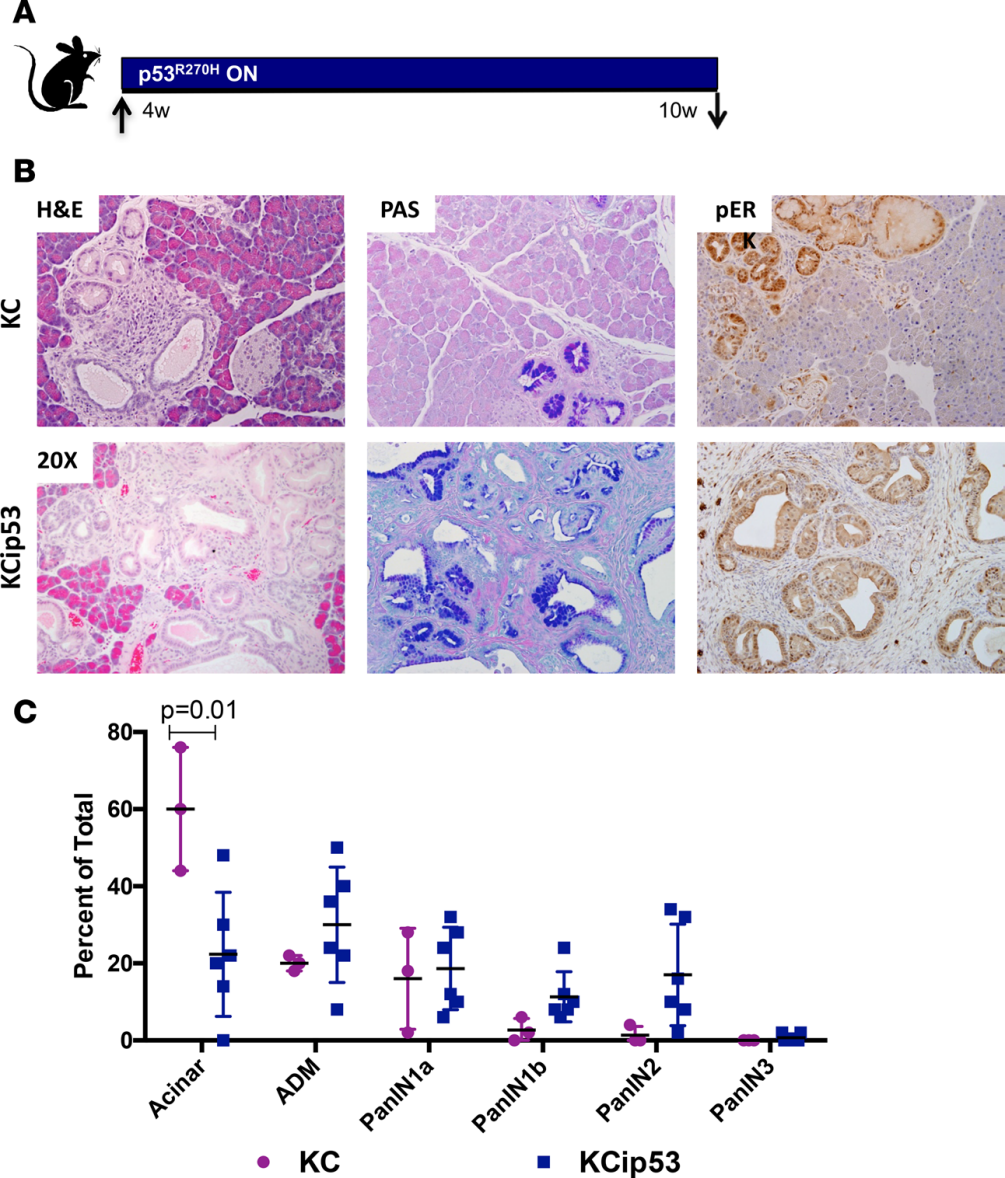
Mutant p53^R270H^ expression promotes PanIN formation. (**A**) Scheme for mouse treatment for early time point analysis. (**B**) H&E, periodic acid–Schiff (PAS), and IHC for pERK in KC and KCip53 animals at 10 weeks of age. (**C**) Quantification of pancreatic pathology in KC and KCip53 animals, analyzed by 2-way ANOVA, presented as mean with SD. *n* = 3–6 animals per genotype. All histology magnification at ×20.

KCip53 and KC littermates were placed on dox at 4 weeks of age, activating expression of mutant p53^R270H^ protein. At 10 weeks of age, pancreata were harvested and subjected to histopathological analysis (Figure 2A). KCip53 pancreata had more PanINs and less normal tissue than pancreata from KC littermates (Figure 2B and quantification in Figure 2C, *n* = 3–6 mice per group). In contrast, in a cohort of KCip53 mice that were never on dox, PanIN progression was indistinguishable from that of KC mice, an indication that expression of the mutant p53 protein was tightly regulated (Supplemental Figure 6, *n* = 3 KC on dox, *n* = 4 KCip53 never on dox).

PanIN lesions in KCip53 animals showed characteristic features, including intracellular mucin accumulation (as indicated by PAS staining) and elevated pERK, indicating elevated MAPK signaling (Figure 2B). Additionally, KCip53 animals had a slight increase in CD45^+^ immune cells (Supplemental Figure 6A) compared with KC controls. STAT3 activation was previously identified as activated downstream of p53^R172H^ in pancreatic cancer cells and a contributing factor in their growth (16). However, immunostaining for the active, phosphorylated form of STAT3 (pSTAT3), revealed no difference in expression of pSTAT3 between KC and KCip53 PanINs, suggesting that a different mechanism might be in play during PanIN formation (Supplemental Figure 6B).

**Figure 3:**
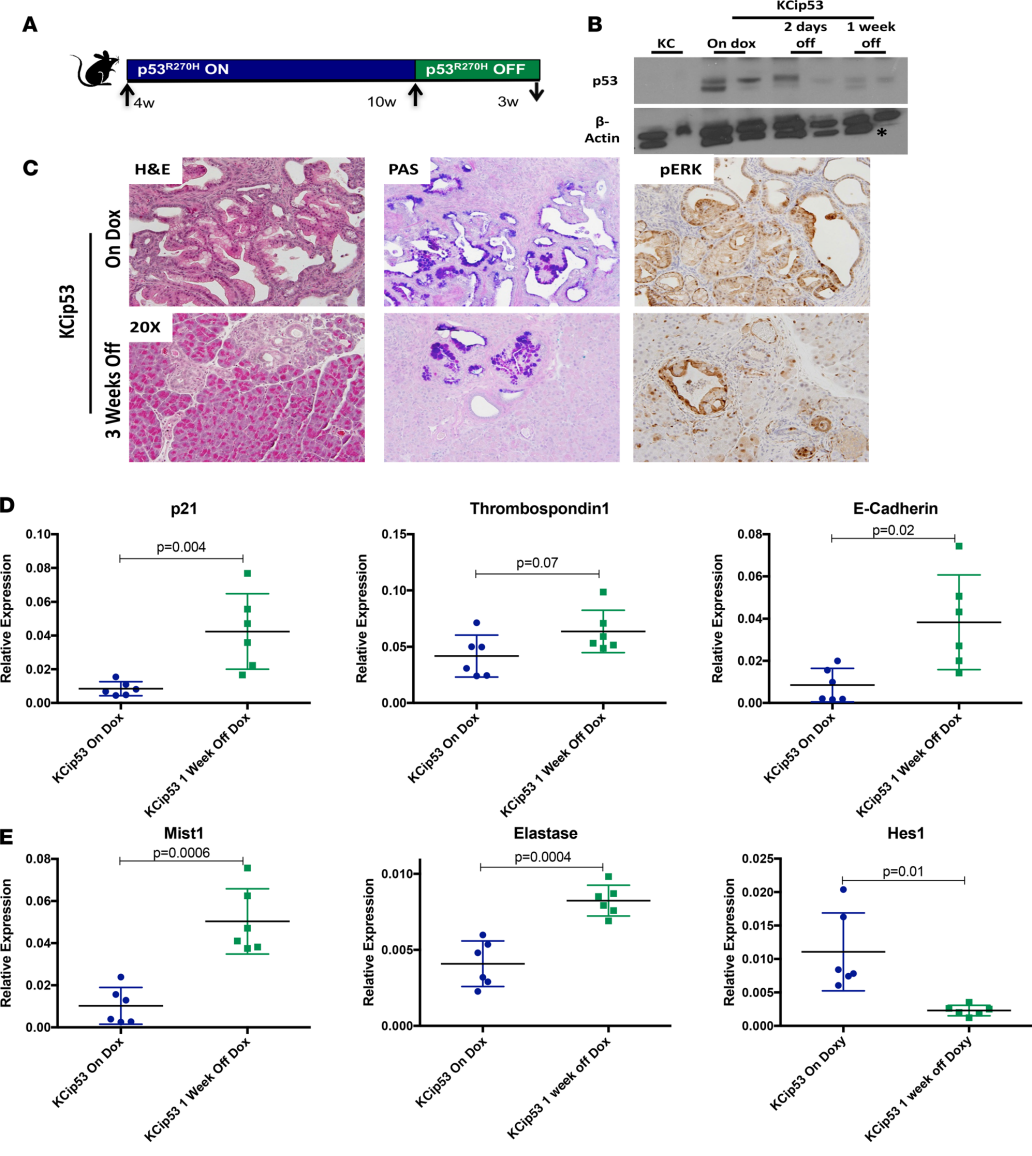
Mutant p53^R270H^ expression is required for PanIN maintenance. (**A**) Scheme for mouse treatment and dox removal. (**B**) Western blot analysis for p53, with each lane representing the lysate from an individual animal. Asterisk indicates a nonspecific band seen in the β-actin blot. (**C**) H&E, PAS, and IHC for pERK in KCip53 animals either on dox or 3 weeks off dox. (**D**) qRT-PCR analysis of pro- and antiapoptotic factors in KCip53 animals on dox and KCip53 animals 2 days off dox. (**E**) qRT-PCR analysis of targets of WT p53 in KCip53 animals on dox and KCip53 animals 1 week off dox. For all qRT-PCR results, unpaired *t* test with Welch’s correction was used, and data are presented as mean with SD. *n* = 3–6 animals per condition. All histology magnification at ×20.

Uniquely, the dox-inducible nature of p53^R270H^ expression allowed us to reverse the expression of mutant p53^R270H^ at different stages of carcinogenesis. We investigated whether mutant p53^R270H^ expression regulated the progression and maintenance of PanINs. Mice were placed on dox at 4 weeks of age for 6 weeks. At that point, the animals were randomized in cohorts that either stayed on dox or were removed from dox. Pancreata from both groups were harvested at 2 days, 1 week, or 3 weeks later (Figure 3A, *n* = 3–6 mice per group). The level of p53 protein accumulation was assessed by Western blotting of whole pancreas lysates. In KC pancreata, p53 protein was undetectable. In KCip53 mice on dox, we observed p53 accumulation, which decreased upon dox removal (Figure 3B). We then compared pancreas histology across the different groups. KCip53 mice that were continuously maintained on dox had extensive PanINs and limited acinar clusters. In contrast, pancreata from mice that were taken off dox for 3 weeks after the initial 6-week dosing period had limited, low-grade lesions and large areas of normal acinar clusters (Figure 3C). Within lesions, PAS and pERK1/2 staining was present in both on- and off-dox pancreata, but fewer positive lesions were observed in the animals taken off dox (Figure 3C). These data are consistent with continuous expression of mutant p53^R270H^ being required for maintenance of PanIN lesions and their continued progression.

Given the dramatic differences seen at the 3-week time point after inactivation of mutant p53, we assessed the effects at 1 week or 2 days after removing dox and the associated mutant p53 expression. No major histological changes were apparent at these earlier time points (Supplemental Figure 7, *n* = 3–6 mice per genotype and time point). We performed qRT-PCR for selected p53-regulated target genes in samples from both cohorts over time. Animals that had been off dox for 1 week had higher levels of WT p53–regulated target gene expression, including for *Cdkn1a* (known commonly, and designated here, as p21), *Thrombospondin1* (*Thbs1*) and *E-Cadherin* (*Chd1*), compared with KCip53 animals maintained on dox (Figure 3D). Acinar markers such as *Bhlha15* (known commonly as Mist1) and *Elastase* were similarly upregulated following inactivation of mutant p53. In contrast, *Hes1*, which is expressed during acinar de-differentiation (17), was downregulated (Figure 3E). To determine whether inactivation of mutant p53 induced cell death, we evaluated the ratio of proapoptotic to antiapoptotic factors; our results showed no statistically significant differences between the two groups of animals (data not shown). By IHC, we determined that E-cadherin, an epithelial marker that is downregulated during carcinogenesis, was higher in animals lacking mutant p53 expression (Supplemental Figure 8). Together, our data indicate that during the early stages of carcinogenesis, mutant p53 sustains tumor progression, and are consistent with the notion that these animals retain some WT function of this tumor suppressor.

### Expression of mutant p53^R270H^ is not required to bypass the oncogenic stress induced by activated Kras^G12D^.

**Figure 4:**
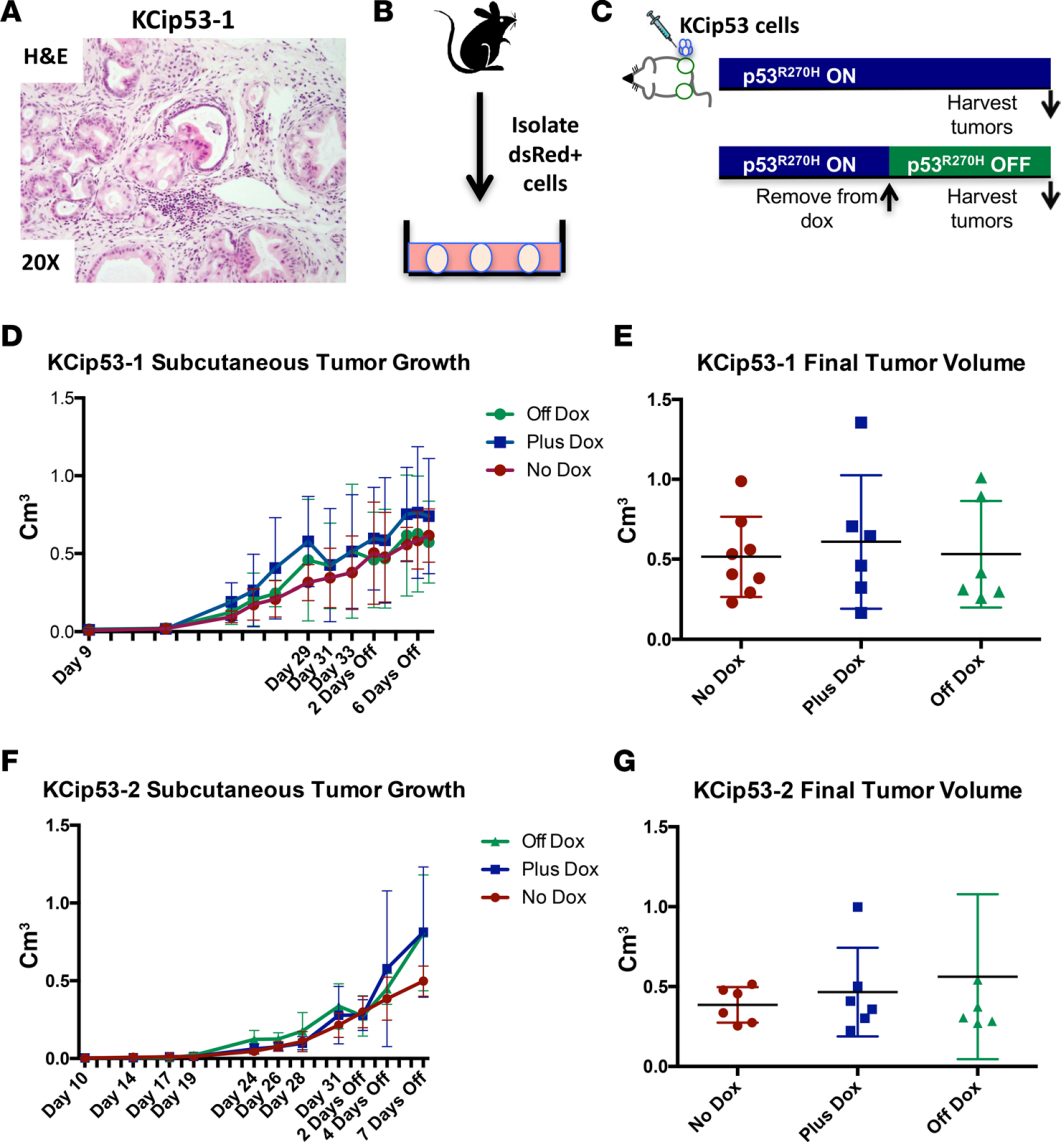
Expression of mutant p53^R270H^ is not required to bypass the oncogenic stress induced by activated Kras^G12D^. (**A**) H&E of primary tumor from the animal from which cell line KCip53-1 was generated, shown at ×20 magnification. (**B**) Scheme of cell line generation from KCip53 animals. (**C**) Scheme for subcutaneous tumor growth assay. (**D**) Subcutaneous tumor growth curve and (**E**) final tumor volume for KCip53-1 cell line. (**F**) Subcutaneous tumor growth curve and (**G**) final tumor volume for KCip53-2 cell line. Data analyzed by 2-way ANOVA, no significantly different results in data shown in this figure. Data are presented as mean with SD. *n* = 6–8 tumors per condition, experiment repeated 2–3 times for each cell line.

We next sought to determine whether continued expression of mutant p53^R270H^ was required for survival and growth of pancreatic cancer cells. As described above, KCip53 mice maintained on dox treatment developed invasive, metastatic tumors with variable latency (Figure 1, C and E). The disease burden in individual animals is heterogeneous, complicating tumor burden analysis at later ages. To study a cohort of mice carrying genetically identical tumors in which we could modulate mutant p53 expression, we established several primary cell lines from cancer-bearing KCip53 mice (Figure 4A). To establish these cell lines, we generated single-cell suspensions from the original primary tumors and sorted the cells by flow cytometry for expression of dsRed, as a surrogate marker for cells expressing mutant p53^R270H^ (Supplemental Figure 9A). The cell lines were then propagated in culture in the presence of dox (Figure 4B). We performed qRT-PCR to study expression of selected p53-regulated target genes, with the goal of determining how changes in expression of mutant p53 affected p53 transcriptional activity in the cells. In cells treated with dox, we saw a reduction in the expression of the WT p53 target genes *Cdkn1a* (AKA p21) and *Thrombospondin1*. Conversely, *Vimentin*, a marker of epithelial-mesenchymal transition (EMT) linked to mutant p53 (18) was downregulated when dox was removed to abrogate p53^R270H^ expression (Supplemental Figure 9B, *n* = 3).

We injected KCip53 cells subcutaneously into immunocompromised (NSG) mice and tracked tumor growth over time by caliper measurement. For each cell line, we included 3 conditions: (i) animals never given dox (no mutant p53^R270H^ expression; termed “no-dox”); (ii) animals always given dox (continued mutant p53^R270H^ expression; “plus-dox”); and (iii) animals started on dox and then removed once tumors had become palpable (termed “off-dox”) (Figure 4C, *n* = 6 or more tumors per group; experiment performed at least twice per cell line). Surprisingly, no differences in tumor growth were observed among these 3 groups for the KCip53-1 and KCip53-2 cell lines (Figure 4, D and F), and there was no difference in final tumor volume or weight among the groups (Figure 4, E and G), indicating that mutant p53^R270H^ expression was not required, at least for growth, at late stages of carcinogenesis.

In human pancreatic cancer samples, mutations in p53 are often accompanied by loss of heterozygosity and consequent inactivation of the WT protein (19). To investigate the status of the WT p53 alleles in KCip53 tumor cell lines, we PCR-amplified the region surrounding exons 7 and 8 of p53 in one KPC cell line (20) and two KCip53 cell lines. In the KPC cells, we amplified a 500-bp band consistent with the placement of the primers across an intron (see scheme in Supplemental Figure 10A). In KCip53 cells, we did not amplify the WT band, but only a shorter band (250 bp), consistent with the cDNA inserted in the transgene. Sequencing of the PCR products confirmed that only the mutant p53 DNA was present on these cells, while the WT sequence was lost (Supplemental Figure 10B).

Histological analysis of the tumors derived from KCip53 cells with or without dox revealed no differences in the prevalence of epithelial cells within the tumors, as measured by expression of CK19, nor in the levels of proliferation (Ki67) or apoptosis (cleaved caspase-3) (Supplemental Figure 11, A–C). To verify the modulation of *Trp53^R270H^* gene expression following dox removal, we performed immunostaining for dsRed. We did not observe any dsRed staining in the tumors from the no-dox cohort. In the plus-dox cohort, we observed heterogeneous accumulation of dsRed, similar to our observations in PanINs. Fewer dsRed-expressing cells were observed in tumors from the cohort removed from dox (off-dox). Hence, dox removal reduced the number of cells expressing the transgene, although a few cells appeared to have escaped regulation and continued to express the transgene in the absence of dox (Supplemental Figure 11D). Quantification of dsRed expression revealed a significantly higher number of cells per high-power field in the dox-treated cohort (Supplemental Figure 11D).

**Figure 5:**
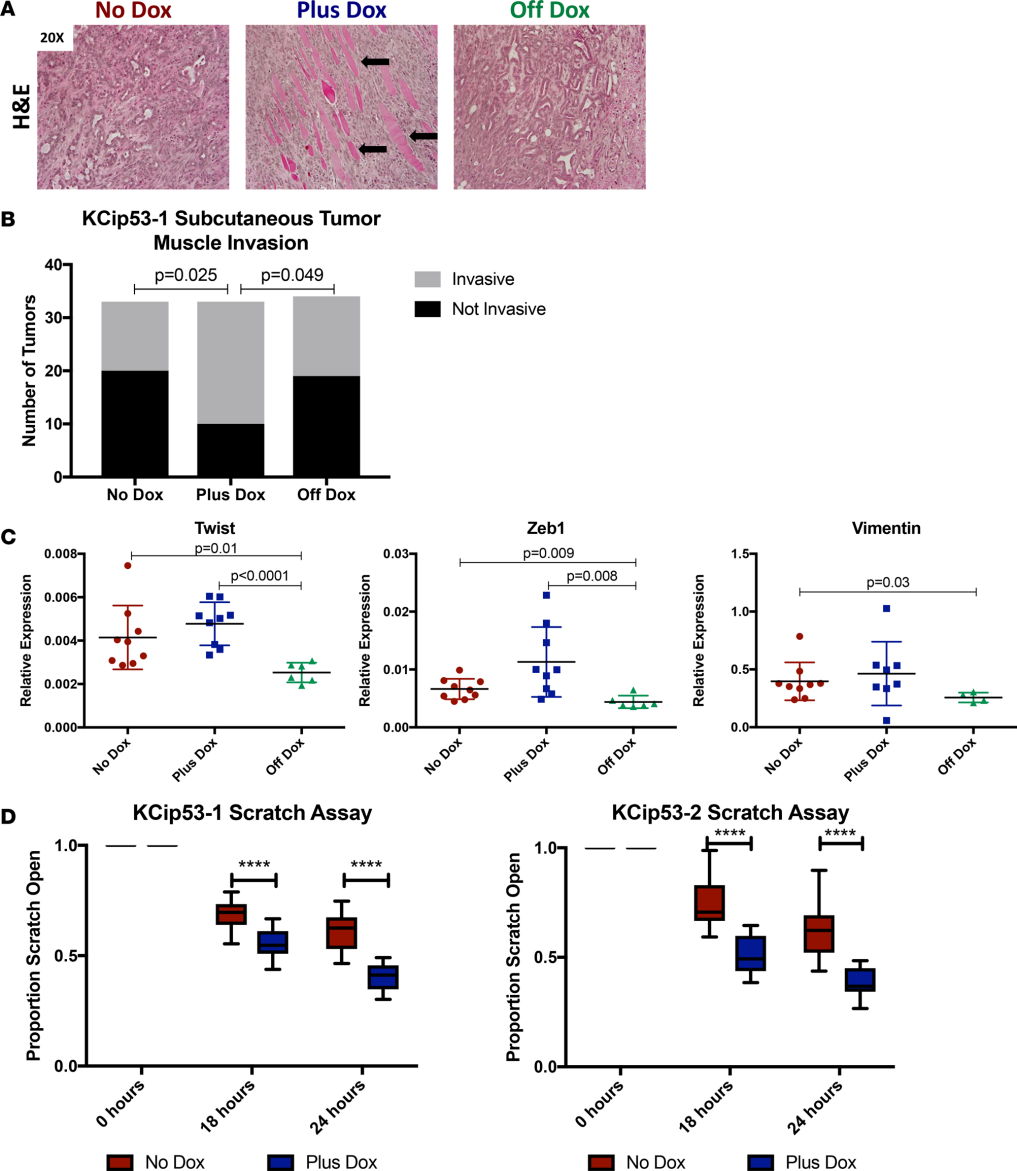
Mutant p53^R270H^ expression promotes cell invasion and migration. (**A**) H&E analysis of final subcutaneous tumors grown from KCip53-1 cells, shown at ×20 magnification. Arrows indicate some muscle fibers within tumor. (**B**) Quantification of percentage of final tumors that have invasion into muscle by H&E. *n* = 33 or 34 tumors per group. Analyzed using Fisher’s exact test. (**C**) qRT-PCR analysis of EMT-associated gene expression in final subcutaneous tumors grown from KCip53-1 cells. *n* = 6–9. For all qRT-PCR results, unpaired *t* test with Welch’s correction was used, and data are presented as mean with SD. (**D**) Scratch assay in KCip53-1 and KCip53-2 cell lines, shown as percentage original of scratch closed at specified time points, analyzed using multiple unpaired *t* test comparisons, represented as mean with SD. *n* = 18 scratch points analyzed per condition. ****P < 0.0001.

Interestingly, comparison of the histology of the tumors from the 3 groups showed differences in epithelial cell morphology. We observed increased muscle invasion in the plus-dox group (Figure 5A, arrows indicate muscle invasion). Immunostaining for CK19 highlighted smaller cells with sarcomatoid features in the same group (Supplemental Figure 11A). Quantification of the phenotype revealed muscle invasion in 39.3% of tumors in mice that were never on dox, and 69.7% of tumors always on dox, suggesting that mutant p53^R270H^ expression promotes tumor invasion into the muscle layer (Figure 5B, *n* = 33 or 34 tumors per group). We then measured the expression of genes associated with EMT, namely *Zeb1*, *Vimentin*, and *Twist1*, by qRT-PCR. While the expression of these genes was generally higher in tumors that maintained expression of mutant p53^R270H^, the difference was not statistically significant (Figure 5C). Since the interpretation of the in vivo data is confounded by differences in the microenvironment, we used an in vitro system for functional studies. For this purpose, we performed scratch assays using KCip53 cell lines. After growing the cells in the presence or absence of dox, we scratched the plate and monitored time to scratch closure. In both KCip53-1 and KCip53-2 lines, cells grown with dox had a shorter time to scratch closure, consistent with an increased migration potential (Figure 5D, *n* = 18 scratch points analyzed per condition). In previous studies, the ability of mutant p53^R172H^ to promote invasion and metastasis of pancreatic cancer cells carrying this specific mutation was mediated by mutant p53–mediated expression of *PDGFRβ* (21). However, we did not observe any difference in PDGFRβ levels by IHC in subcutaneous tumors grown from KCip53-2 cells (Supplemental Figure 12A). Additionally, qRT-PCR for *Pdgfrβ* in KCip53 pancreata 3 weeks off dox was actually slightly higher than in animals on dox (Supplemental Figure 12B). Together, these data indicate that p53^R270H^ may control invasive behavior through a different mechanism than p53^R172H^.

Given the changes in in vitro migration, and the increased muscle invasion observed in subcutaneous models, we implanted KCip53-1 cells orthotopically into the pancreata of immunocompromised mice to determine whether mutant p53^R270H^ expression conferred increased metastatic potential, as previously described for p53^R172H^ (12, 21). As in the subcutaneous experiment, we divided the animals into 3 cohorts (no-dox, plus-dox, and off-dox). We then measured tumor growth over time by MRI, starting 2 weeks after cell implantation and continuing for 3 weeks, when the tumors were harvested (Supplemental Figure 13A, *n* = 3–4 animals per group). Similar to the subcutaneous experiment, there was no difference in tumor growth rate, final tumor volume, or metastatic spread between the no-dox and plus-dox groups. Tumors in the cohort taken off dox were slightly smaller (Supplemental Figure 13, B–F). Histology of the tumors across cohorts was similar. These experiments were performed with only a small group of animals, but tumor growth rate and characteristics were consistent with those seen in the subcutaneous tumor growth experiments, suggesting that the phenotype is the same. Here mutant p53^R270H^ can confer increased migration potential to cells in certain contexts, but its expression is not necessary to alleviate the oncogenic stress caused by Kras^G12D^.

### Transcriptional profile of genes activated downstream of p53^R270H^ reveals alterations in cellular metabolism.

**Figure 6:**
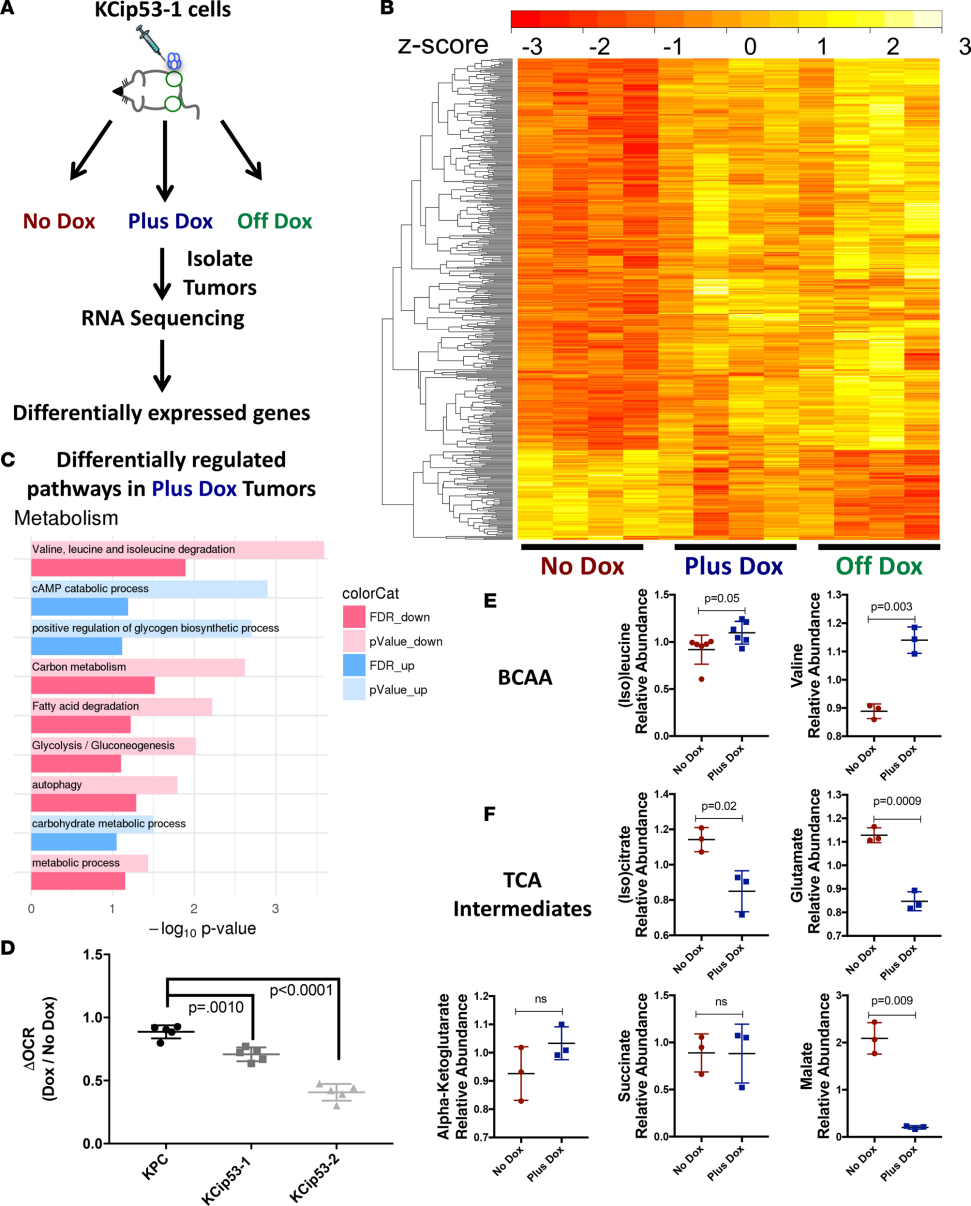
Transcriptional profile of genes activated downstream of p53^R270H^ reveals alterations in cellular metabolism. (**A**) Scheme for subcutaneous tumor growth and RNA collection for RNA-seq. *n* = 4 samples per group. (**B**) Heatmap showing differential gene expression in tumor groups. (**C**) Pathway analysis of gene expression differences in no-dox and plus-dox conditions. (**D**) Change in OCR levels as measured by mitochondrial stress test in KPC, KCip53-1, and KCip53-2 cells with or without dox, analyzed using 1-way ANOVA. *n* = 5 replicates per condition. Difference of levels of (**E**) branched chain amino acids and (**F**) TCA intermediates in KCip53-1 cells grown with or without dox, analyzed using unpaired *t* test with Welch’s correction, shown as mean with SD. *n* = 3 samples per group.

To understand the global effects of expressing p53^R270H^, we performed RNA sequencing. We subcutaneously injected KCip53-1 cells into NSG mice and divided the injected mice into 3 experimental groups, as described above: no-dox, plus-dox, and off-dox (Figure 6A). We then extracted RNA from bulk, unsorted tumors. RNA sequencing analysis revealed marked differences in gene expression profiles among the 3 treatment groups (*n* = 4 tumors/group), with the most significant gene expression signature differences between the group that had no mutant p53^R270H^ expression and the group of tumors always expressing mutant p53^R270H^ (Figure 6B). Intriguingly, the gene profile established by p53^R270H^ expression did not reverse upon inactivation of the mutant (Figure 6B and Supplemental Table 1).

To analyze the data, we performed pathway analysis and assessed groups of genes whose expression changed between the no-dox and plus-dox groups. We also observed changes in cell signaling, cell fate, extracellular matrix modeling, and cell motility (Supplemental Figure 14 and Supplemental Table 2). Mutant p53^R270H^ expression also reduced apoptotic pathways, while conversely upregulating signaling pathways that might reflect the increased growth potential of these cells (growth factor activity, regulation of the ERK1/2 cascade, and regulation of the insulin receptor signaling pathway). Further, mutant p53^R270H^ expression correlated with increased ECM remodeling pathways, cell mobility pathways, and activation of Rho pathway activities that control cytoskeletal dynamics, all linked with cell invasion and migration. Among the pathways altered by mutant p53 expression, we identified several that suggested changes in metabolism (Figure 6C), including regulation of the metabolism of amino acids, carbon sources, fatty acids, and autophagy. Given our experimental design, the gene expression differences could reflect changes within the tumor cells themselves or in the surrounding stroma in response to epithelial p53 expression.

To functionally investigate whether p53^R270H^ regulates pancreatic ductal adenocarcinoma (PDA) metabolism, and determine which specific changes were relevant to the epithelial cell compartment, we analyzed intercellular metabolites from primary KCip53 cells with or without dox treatment by targeted liquid chromatography–mass spectrometry–based (LC-MS–based) metabolomics (scheme in Supplemental Figure 15A, *n* = 3 samples per condition). As the pathway analysis suggested, we observed profound changes across the metabolome (Supplemental Figure 15B and Supplemental Table 3). Among these, and consistent with the pathway analysis, we found that intracellular branched chain amino acid (BCAA) levels were elevated (Figure 6E), which could reflect lower catabolism. BCAAs can be used as a carbon source to fuel the tricarboxylic acid (TCA) cycle in the mitochondria. Indeed, we also observed lower levels of several metabolites in the TCA cycle (Figure 6F and Supplemental Figure 15C), which could again reflect lower mitochondrial activity. Thus, to assess bioenergetic activity directly, we used a Seahorse instrument to measure changes in mitochondrial metabolism and glycolysis, as read out by oxygen consumption rate (OCR) and extracellular acidification (ECAR), respectively. This was performed for primary KCip53 cell lines and a primary cell line isolated from a mouse Pdx-Cre;Kras^LSL-G12D^;p53^R172H^ (KPC) tumor. The latter do not depend on dox for their mutant p53 expression and served as controls. We observed a decrease in OCR in the KCip53 cell lines, but not in the KPC line, upon dox treatment (Figure 6D and Supplemental Figure 16A, *n* = 5 samples per condition). Changes in p53 levels in KCip53 cell lines upon dox treatment, but not KPC were confirmed by Western blot analysis (Supplemental Figure 16B). These functional data provide direct evidence that mutant p53^R270H^ expression reduces mitochondrial activity, which may result from decreased BCAA metabolism.

### Inactivation of mutant p53^R270H^ function sensitizes tumors to MEK inhibition.

**Figure 7:**
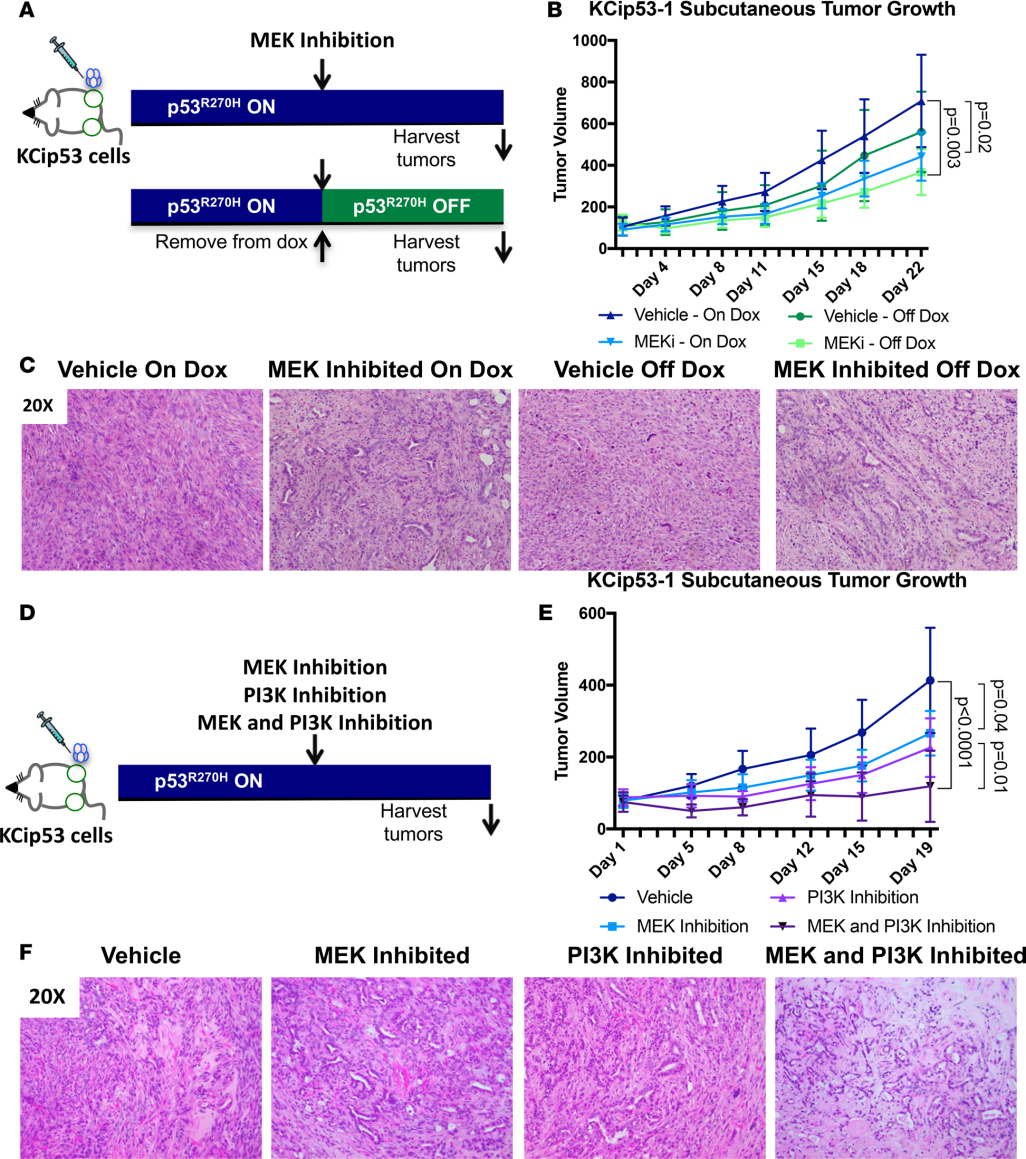
Inactivation of mutant p53^R270H^ function sensitizes tumors to MEK inhibition. (**A**) Scheme for subcutaneous tumor growth and drug treatment using KCip53-1 cells in MEK inhibition and p53^R270H^ on and off groups. (**B**) Subcutaneous tumor growth curve for treatment groups using KCip53-1 cells. MEKi, MEK inhibition. (**C**) H&E analysis of final tumors. (**D**) Scheme for subcutaneous tumor growth and drug treatment using KCip53-1 cells in MEK inhibition and AKT inhibition groups. (**E**) Subcutaneous tumor growth curve for treatment groups using KCip53-1 cells. (**F**) H&E analysis of final tumors. *n* = 10 tumors per group for MEK inhibition, *n* = 8 tumors per group for MEK and PI3K inhibition. Histology shown at ×20 magnification. Tumor growth analyzed by 2-way ANOVA, shown as mean with SD.

Upward of 90% of pancreatic cancers harbor oncogenic Kras mutations, but no targeting agents are currently available (for review, see ref. 22). Attempts to target downstream effector pathways activated by oncogenic Kras, such as PI3K/AKT or MAPK signaling (22) — both important in pancreatic carcinogenesis (23–27) — have similarly been unsuccessful (28). To determine whether inactivation of *Trp53^R270H^* expression, and subsequent reactivation of WT p53, sensitized pancreatic cancer cells to MAPK or AKT inhibition, we designed a set of experiments. We injected NSG mice subcutaneously with KCip53-1 cells. Initially, all the mice were kept on dox to express mutant p53. In our first experiment, we tested for MEK sensitivity. Once the tumors were palpable (approximately 2 weeks after injection), we subdivided the mice into 4 groups: (i) on dox with vehicle; (ii) on dox with MEK inhibitor (PD325901, 5 mg/kg administered daily by oral gavage); (iii) off dox with vehicle; and (iv) off dox with MEK inhibitor (Figure 7A, *n* = 5 mice per group, two tumors implanted per mouse). We measured tumor volume over time by MRI. Similar to previous observations (20, 23), MEK inhibition slowed but did not reverse tumor growth, whether or not mutant *Trp53^R270H^* was expressed. Abrogation of mutant p53 expression slightly decreased tumor growth, while MEK inhibition cooperated with abrogation of mutant p53^R270H^ to decrease tumor growth (Figure 7B). By immunostaining and Western blot analysis, we observed decreased phospho-ERK1/2 (indicating MAPK activity) in the MEK inhibitor–treated samples and increased expression of dsRed (as readout for mutant p53 expression) in samples from mice on dox both with and without drug treatment, as expected (Supplemental Figure 17). We did not observe changes in proliferation (measured by Ki67) or apoptosis (by cleaved caspase-3 staining) in any of the groups, possibly due to the fact that histological analysis was performed long after the start of drug treatment. The MEK-inhibited and off-dox group displayed higher levels of fibrosis, reflecting the slowing of cell growth in these tumors (Supplemental Figure 17). Histological characterization of the tumors showed changes in cellular architecture, with more ductal structures and a less-sarcomatoid appearance in the tumors from MEK-inhibited groups (Figure 7C). We, and others, have previously shown that MEK signaling regulates epithelial growth and differentiation as well as aspects of stroma activation and remodeling (23, 24, 26, 29). Thus, the changes in the accumulation of the stroma and tissue architecture might reflect a combination of cell-autonomous and non-cell-autonomous effects.

Following the MEK inhibition studies, we pursued efforts to study combined inhibition of MEK and PI3K, an upstream regulator of AKT, in mice harboring the KCip53 tumors. We injected NSG mice subcutaneously with KCip53-1 cells, and animals remained on dox, with mutant p53^R270H^ expression, throughout the experiment. Once the tumors were palpable, we started drug treatment in all 4 groups: vehicle, MEK inhibition, PI3K inhibition, and dual MEK and PI3K inhibition (Figure 7D, *n* = 4 animals per group, two tumors implanted per animal). The MEK inhibitor PD325901 was administered orally, once daily at a dose of 5 mg/kg. The PI3K inhibitor ZSTK-474 was administered orally once daily at a dose of 100 mg/kg. Tumor growth was assessed using MRI for exact volume measurements. As expected, tumors in the control group grew the fastest, while single MEK or PI3K inhibition had a modest inhibitory effect on growth, which was statistically significant only for PI3K inhibition. The combination of MEK and PI3K inhibition potently inhibited tumor growth, with no increase in tumor volume seen in 4 of 8 tumors, although no regression of tumors was evident (Figure 7E). We confirmed MEK and PI3K inhibition in the respective treatment groups by Western blot analysis, observing downregulation of pERK1/2 in the MEK inhibitor–treated groups and reduced pAKT in the PI3K inhibitor groups (Supplemental Figure 18). Interestingly, while vehicle-treated tumors showed sarcomatoid histology, consistent with the histology of this cell line, inhibition of either MEK or PI3K led to an increase in ductal structures surrounded by fibrotic stroma (Figure 7F). In the dual MEK/PI3K inhibitor–treated samples we observed ductal structures surrounded by stroma with lower cellularity than any of the other groups. Proliferation, measured as Ki67-positive nuclei, was lowest in the dual inhibitor–treated tumors. In contrast, we did not observe changes in apoptosis (cleaved caspase-3 expression) in any of the groups, although it is possible that we missed an earlier wave of cell death (Supplemental Figure 18). Thus, restoration of WT p53 function at least partially sensitized pancreatic tumor cells to MEK inhibition; however, expression of the specific Trp53^R270H^ mutant did not change response to combined MEK and PI3K inhibition, compared with other pancreatic cancer cell lines. These novel KCip53 cell lines constitute a platform for testing the effects of WT p53 restoration in combination with targeted inhibitors.

## Discussion

In human tumors, the majority of TP53 mutations in pancreatic cancer lead to the expression of a missense mutant p53 protein that can still oligomerize with WT p53 but fails to function as a sequence-specific DNA transcription factor (4, 5, 30). Consistent with the human data, expression of mutant Kras in the pancreas is sufficient to initiate carcinogenesis, while *Trp53* mutations have little effect on their own but promote carcinogenesis in the presence of oncogenic Kras (10, 11). Of the spectrum of human mutations, two types have been studied in mouse models: p53 deletion and a substitution of histidine for arginine at codon 172 of the mouse protein (p53^R172H^), ortholog to R175H in the human p53 protein. Deletion of one or both alleles of p53, in the presence of oncogenic Kras, leads to invasive pancreatic tumors with short latency and high penetrance (31). In the commonly used KPC model, p53^R172H^ is expressed alongside oncogenic Kras; this model has slightly longer latency to invasive disease and, at least in some reports, increased metastatic potential (11). However, these models introduce both Kras and p53 mutations at embryonic stages, and do not mimic the sequence of mutations that occur in human tumors. In this study, we reanalyzed a public sequencing database (COSMIC) to determine the prevalence of individual TP53 mutations in human pancreatic cancer. The codon encoding for amino acid 273 of the human protein was the most frequent mutation site; at this site, the most common mutation was R273H. Interestingly, this mutation is considered a p53 “hotspot” mutation, and is one of the most common mutations in human tumors of all types (32). Since this specific mutation had not been expressed in the pancreas in mice expressing mutant Kras, we set out to model it. Thus, we expressed *Trp53^R270H^*, the mouse ortholog to human *TP53^R273H^*, in the KC mouse model of pancreatic cancer. The KCip53 mouse combines expression of oncogenic Kras with *Trp53^R270H^*, and it is designed to have two unique features: (i) *Trp53^R270H^* expression in this model is inducible, thus allowing sequential activation of mutant genes; and (ii) *Trp53^R270H^* expression is reversible, and thus inactivation of its expression and, consequently, restoration of WT function — at least until loss of heterozygosity (LOH) for p53 occurs spontaneously — can be regulated at will. Similar to p53^R172H^, p53^R270H^ synergizes with oncogenic Kras to promote pancreatic carcinogenesis. However, a comparison between KPC mice, expressing p53^R172H^, and the KCip53 model revealed slower progression to malignancy in the latter. Different explanations are possible for this finding, including the later time of activation of mutant p53 (in adult mice rather than in embryogenesis), as well as the fact that the KCip53 model retains two copies of the WT allele. Interestingly, KCip53 mice develop a highly metastatic tumor, which is consistent with a role for p53 in inducing EMT, one of the mechanisms of metastatic disease spread. Further, tumors expressing mutant p53 showed increased intramuscular invasion in the subcutaneous setting, reflecting our observation in vitro, where mutant p53 promotes cellular migration.

A unique feature of the KCip53 model is the reversible nature of mutant p53 expression, which allows us to restore WT p53 function at will, as well as to understand mutant-specific phenotypes. In PanIN lesions, expression of the mutant was continuously required for progression, and inactivation of mutant p53 led to an increase in expression of WT p53 target genes, such as p21, that inhibit growth. The mutant protein also facilitated survival of epithelial cells exposed to Kras-driven oncogenic stress, and its inactivation increased the ratio of proapoptotic to antiapoptotic factors. Our data support a model whereby PanIN progression to cancer is unlikely if WT p53 is retained, as is the case in the majority of human PanINs (3). Conversely, acquisition of mutations in the *Trp53* gene allows bypassing this early arrest and progression to cancer. Unique to our model, inactivation of mutant p53 inhibits progression even once the neoplastic process has begun. Similar to our findings, restoration of WT p53 in liver and lung tumors limits cancer progression by inducing apoptosis (33–35). In those studies, WT p53 expression was restored to autochthonous tumors that had developed in the absence of p53 expression in mutant *Ras*-driven liver or lung tumor models. In each of these models, WT p53 restoration led to tumor regression. For this reason, restoration of WT p53 function has been hypothesized as a potential therapeutic approach — an attractive prospect given that p53 is lost or mutated in the vast majority of human malignancies (32). Each of the p53 restoration studies relied on models that had lost p53 function. Given that p53 mutation, instead of loss of function, is common in human cancer development, the KCip53 animals provide a unique system to test questions of WT p53 restoration in tumor therapy.

Our PanIN studies supported the idea that restoration of WT p53 protein limits tumor progression. Surprisingly, in invasive tumors, mutant p53^R270H^ inactivation had no apparent effect on tumor growth, at least in the transplantation setting, indicating that other mechanisms might compensate to avoid oncogene-induced senescence. Sequencing analysis revealed that the tumor cells had lost their WT copies of p53, possibly explaining their continued growth even upon inactivation of the mutant protein. Nevertheless, other fundamental tumor characteristics depended on continuous expression of the mutant p53^R270H^ protein. RNA-seq analysis revealed profound changes induced by mutant p53 expression. However, inactivation of p53^R270H^ in established tumors failed to restore the baseline gene expression pattern. In other words, expression of mutant p53 altered the transcriptome of the tumor cells, but its removal failed to restore transcription to the baseline pattern. This finding might explain the lack of dependence on mutant p53 of the KCip53 cell lines, although selective pressure to retain transgene expression might play a role as well. Pathway analysis illuminated many processes that are perturbed by expression of mutant p53^R270H^, several of which have been previously identified as important in pancreatic cancer. Among these, we found changes in the MAPK and Rho family signaling pathways, both critical for pancreatic tumorigenesis (26, 36–38). Mutant p53^R172H^ expression in pancreatic cancer cells induces metastasis, an effect that was attributed to activation of PDGFRβ (21). Analysis of the RNA-seq data revealed induction of *Pdgfrβ* upon expression of p53^R270H^, albeit to a modest extent. IHC staining for PDGFRβ expression in subcutaneous tumor tissue revealed high levels of PDGFRβ expression even in no-dox tumors, which may explain the only modest increase in the plus-dox condition.

Conversely, cellular metabolism was profoundly altered by expression of mutant p53^R270H^. The complex role of p53 in the regulation of cellular metabolism is an area of active investigation, and it is increasingly being understood that p53 directly influences multiple metabolic pathways (39). An interesting observation, based on pancreatic cancer patient–derived xenografts, is that lack of p53 function sensitizes tumors to inhibition of lactate dehydrogenase (40), indicating a link between p53 function and glycolysis in pancreatic cancer. It has also been suggested that p53 function can play a role in the regulation of autophagy in pancreatic tumorigenesis, (41, 42). Here, using gene expression profiles, we identified that the glycolysis and autophagy pathways are altered as a result of p53^R270H^ expression. These results, coupled with the observed decrease in mitochondrial activity when mutant p53^R270H^ is expressed, suggest that p53 mutations play an integral role in the metabolic wiring of pancreatic cancer cells. Supporting this notion, changes in metabolic phenotype in pancreatic cancer change when the cells undergo EMT (43). The potential causal relationship between EMT and metabolic changes induced by mutant p53 remains to be elucidated.

Interestingly, pathway analysis of our RNA-seq data revealed downregulation of BCAA metabolism upon expression of p53^R270H^. Further, we confirmed this finding by measuring metabolite abundance. The incorporation of BCAA-derived carbon into the TCA cycle decreases in pancreatic tumors harboring a Kras mutation and p53 LOH, relative to corresponding normal pancreas (44). Similarly, we observed that the BCAA degradation pathway was downregulated upon expression of p53^R270H^, indicating that in this context p53 mutation acts similarly to p53 loss. Taken together, these observations suggest a key role for mutant p53 in shaping the nutrient acquisition of pancreatic cancer cells. Thus, future research into the mechanisms by which p53 mutations shape PDA metabolism might identify potential sensitivities of p53 mutant tumors suitable for therapeutic targeting.

We, and others, have previously shown that pancreatic cancer requires continuous oncogenic Kras activity (45–47). We have also identified several therapeutically actionable mechanisms by which Kras drives the re-wiring of pancreatic cancer metabolism (47, 48). The data presented here represent an initial snapshot into the metabolic landscape of pancreatic cancer as shaped by mutations in the tumor suppressor p53, which may serve to highlight new therapeutic vulnerabilities. While our model uses genetic manipulation to inactivate mutant p53^R270H^ expression in pancreatic cancer, a new generation of conformational drugs has been developed for this purpose (33, 49–53). Our results indicate that those drugs by themselves might have limited effect on pancreatic cancer growth. However, combined targeting of mutant p53 and specific metabolic pathway inhibitions might reveal unexpected vulnerabilities of pancreatic cancer. Identification of ideal combinations and of the appropriate patient population — among the different pancreatic cancer subsets (9, 19, 54–56) — will be essential to move this concept toward the clinic.

## Methods

### Mice.

Mice were housed in specific pathogen–free facilities at the University of Michigan Comprehensive Cancer Center. This study was approved by the University of Michigan Committee on Use and Care of Animals (UCUCA). Ptf1a-Cre, Pdx1-Cre, LSL-Kras^G12D^, LSL-Tp53^R270H^, and R26-rtTa animals have been previously described (10, 11, 45, 57), and TREp53^R270H^ animals were generated at the University of Michigan (E.R. Fearon). Animals were analyzed at various ages, as described in the text. KCip53 animals were not backcrossed and were maintained on a mixed background. Dox was administered in the drinking water (0.2 g/l in a 5% sucrose solution) or chow (1 g/kg) and replaced every 3–4 days. Immunocompromised (NSG) animals were used for all subcutaneous or orthotopic tumor growth experiments. NSG animals of both sexes were ordered from the Jackson Laboratory. Animals were ordered at 6–8 weeks of age, and experiments were initiated within a week of the arrival of mice at the University of Michigan. For subcutaneous tumors, 500,000 cells were injected in a 200-μl solution of 50% Matrigel and 50% RPMI media. Each mouse was implanted subcutaneously with two tumors, and tumor growth was recorded at least 3 times weekly until tumors reached a 1.5-cm diameter. For orthotopic tumors, mice were anesthetized using vaporized isoflurane. 500,000 cells were injected into a 50-μl solution of 50% Matrigel and 50% RPMI media directly into the pancreas. The incision was closed using absorbable sutures and clips. Postsurgical animals were monitored daily for 10 days, and then at least 3 times weekly for the duration of the experiment. For drug treatments, animals were treated with PD325901 at a dose of 5 mg/kg, ZSTK-474 at a dose of 100 mg/kg, or vehicle. Combination treatment was given using the same doses for single drug treatment. All treatments were given by oral gavage once daily.

### IHC.

Histology and IHC studies were performed as previously described (45). Primary antibodies used are detailed in the supplemental material.

### qRT-PCR.

RNA isolation, RT-PCR, and qRT-PCR were performed as previously described (45). *Cyclophilin A* was used as the control housekeeping gene for normalization. Primers used are listed in the supplemental material.

### Histopathological analysis.

Histopathological analysis was performed using H&E-stained sections, with the pathologist being blinded to each animal’s genotype. At least 3 independent animals were analyzed from each group, with a minimum of 50 acinar or ductal clusters being counted from each animal. Five representative, non-overlapping, high-power images were analyzed from each slide, with one slide being analyzed per animal. Each cluster was classified as acinar or PanIN1A, -1B, -2, or -3, based on the classification consensus.

### Western blot analysis.

Protein isolation and Western blot analysis were performed as previously described (45). Antibodies used are detailed in the supplemental materials.

### Cell culture.

KCip53 cell lines were derived from mice of the genotype Ptf1a-Cre;LSL-Kras^G12D^;TREp53^R270H^;R26^rtTa/rtTa^. Pancreata or tumors from these animals were minced with scissors and digested in 1 mg/ml collagenase. Tumor cells were then sorted from these cultures using FACS, sorting for the expression of dsRed. All cells were cultured in RPMI supplemented with 10% FBS and 1% penicillin/streptomycin (Gibco). Dox was administered to cells at a concentration of 2 μg/ml.

### Scratch assay.

Cells were plated in a 6-well plate and grown until confluent. Scratches were created with a pipette tip. Scratches were then measured in the same location at each time point. Distance of scratch closure was measured using ImageJ (NIH).

### MRI.

Mice were anesthetized with 1%–2% isoflurane/air, and body temperature was maintained using a Multistation Temperature Control Unit (Minerve Equipment Veterinaire.) MRI scanning was performed using a 3T translational, cryogen-free, preclinical MRI (MR Solutions; MRS 3000 series) with a quadrature mouse body volume coil. Mice were placed supine in the mouse bed. To reduce respiratory motion, surgical tape was used to secure the mice below the thoracic cavity on the bed. T2-weighted images were acquired using a fast spin echo multi-slice sequence with the following parameters: repetition time (TR)/echo time (TE) = 4,500/34 ms, 8 echo trains, 4 averages, field of view (FOV) = 35 × 35 mm^2^, matrix size = 128 × 128, slice thickness = 2 mm, number of slices = 30, and no gap. Using in-house software, the tumor boundary was manually defined on each slice and then integrated across slices to measure the volume.

### RNA-seq.

Tissue from subcutaneous tumors was collected in lysis buffer for RNA extraction. RNA was isolated using the QIAGEN AllPrep DNA/RNA/miRNA Universal Kit according to the manufacturer’s instructions. PolyA+, non-strand-specific libraries were prepared by the University of Michigan Sequencing Core, and all samples were sequenced on an Illumina HiSeq 4000. RNA-seq data are available at the NCBI’s Gene Expression Omnibus database (GEO GSE106853).

### RNA-seq data analysis.

50-bp, single-end reads were mapped to the mouse reference genome (mm9) using TopHat v 1.4.1 (58). The NCBI RefSeq transcript isoform annotation was condensed to an unstranded, gene-level annotation and quantification performed over unambiguous exonic spans per gene. Gene expression was calculated as RPKM (reads per kilobase million; using exonic, base-wise coverage, normalized by summed lengths of exons and total mapped read count). Differential gene expression was performed using DE2 v1.12.4 (59). Differentially expressed genes were defined as being expressed more than 0.25 RPKM (mean value across all samples in a given comparison), greater than 250 bp in length, changing more than 1.5-fold, and having an adjusted *P* value from DESeq2 of less than 0.1. For cluster analysis and heatmap generation, gene expression values were log_10_ transformed and *z*-score standardized across samples. Hierarchical clustering was performed using Euclidean distance and complete linkage. RNA-seq data are available at the NCBI’s GEO database (GSE106853).

### Metabolic flux assay.

To estimate the rate of glycolysis and mitochondrial respiration, a Seahorse Metabolic Flux Analyzer e96 XF instrument (Agilent) was used according to the manufacturer’s manual. 20,000 cells/well of KPC, KCip53-1, or KCip53-2 cells were seeded in the respective culture media the day prior to the assay. The next day media was exchanged to the Seahorse assay media, containing 25 mM glucose, adjusted to 7.4 pH. The cell plate was allowed to equilibrate for 1 hour in a non-CO_2_, 37°C incubator, and following 3 sequential measurements for the basal respiration, a Seahorse XF Mito Stress assay (Agilent) was performed by injections of 1 μM oligomycin, 1 μM FCCP, 0.5 μM rotenone/0.5 μM antimycin A. All the chemicals were obtained from Sigma-Aldrich. The cell number adjustment was performed using CyQuant NF (Thermo) after the assay. The data are presented as mean and SD.

### Label-free targeted metabolomics and data analysis.

PDA cells were plated in triplicate and treated with dox containing medium or normal RPMI with 5% FBS for 3 days. The medium was removed and the cell lysate harvested with ice-cold 80% MeOH. The soluble metabolite fractions were cleared by centrifugation, dried via SpeedVac (Thermo Fisher), then resuspended in a 50:50 MeOH/H_2_O mixture for LC–MS analysis. We performed label-free targeted metabolomics using in-house LC-MS to measure more than 200 metabolites. The bioinformatic data analysis was done using R/Bioconductor (http://www.bioconductor.org). Further details are provided in the supplemental methods.

### Statistics.

All data is represented as mean with SD. For analysis of qRT-PCR, unpaired *t* test with Welch’s correction was used. Subcutaneous tumor growth experiments were analyzed by 2-way ANOVA. A *P* value of less than 0.05 was considered significant for all analyses. Statistical analysis was performed in GraphPad Prism software.

### Study approval.

All animal studies were approved by the University of Michigan UCUCA.

## Author contributions

HKS, SG, ERF, and MPM conceived the study concept and design; HKS, JZ, ACD, TF, CJH, BM, ADV, YF, CKS, WY, CE, JPM, ML, SG, HJL, IK, SAK, and DMK acquired and analyzed data; MPM directed the study; HKS wrote the initial draft of the manuscript; HKS, CJH, CAL, SG, ML, ERF, and MPM edited and finalized the manuscript.

## Supplementary Material

Supplemental data

Supplemental Table 1

Supplemental Table 2

Supplemental Table 3

## Version 1 01/25/2018

Electronic publication
