# Supplementary material for: Mutant p53R270H drives altered metabolism and increased invasion in pancreatic ductal adenocarcinoma
Source: JCI Insight. 2018 Jan 25;3(2):e97422. doi: 10.1172/jci.insight.97422 (PMC5821189; doi:10.1172/jci.insight.97422)
Supplement: Supplemental data [file jciinsight-3-97422-s001.pdf]

Antibodies used in this study

Antibody	Supplier	Catalog Number	IHC Dilution	IF Dilution	Western Blot Dilution
P53	Vector Labs	VP-P956	1:500		
P53	Novacastra	P53-CM5P-L	1:1000		1:5000
RFP	Rockland	600-401-379	1:300	1:300	
pERK1/2	Cell Signaling	4370L	1:100		1:1000
tERK1/2	Cell Signaling	4695S			1:1000
pAKT	Cell Signaling	4060L			1:1000
tAKT	Cell Signaling	9272S			1:1000
CD45	BD Biosciences	553076	1:200		
pSTAT3	Cell Signaling	9131	1:100		
CK19 (TROMAIII)	Iowa Developmental Hybridoma Bank	-	1:100	1:100	
Ki67	Vector Laboratories	VP-RM04	1:100		
Cleaved Caspase 3	Cell Signaling	9961	1:300		
β-Actin	Santa Cruz	SC-69879			1:2500

Primers Used in this study

Gene	Forward Primer	Reverse Primer
Cyclophilin	TCACAGAATTATTCCAGGATTCATG	TGCCGCCAGTGCCATT
Bax	AAACTGGTGCTCAAGGCCCT	AGCAGCCGCTCACGGAG
Bcl-2	CCGGGAGAACAGGGTATGATAA	CCCACTCGTAGCCCCTCTG
Bcl-X	GGTCGCATCGTGGCCTTT	TCCGACTCACCAATACCTGCAT
Bak	TATTAACCGGCGCTACGACAC	CTTAAATAGGCTGGAGGCGATCTT
Cdkn1a	CCTGGTGATGTCCGACCTG	CCATGAGCGCATCGCAATC

(p21)		
Thbs1	GGGGAGATAACGGTGTGTTTG	CGGGGATCAGGTTGGCATT
Cdh1 (E-Cadherin)	CAGCCTTCTTTTCGGAAGACT	GGTAGACAGCTCCCTATGACTG
Hey1	GCGCGGACGAGAATGGAAA	TCAGGTGATCCACAGTCATCTG
Igf2	GTGCTGCATCGCTGCTTAC	ACGTCCCTCTCGGACTTGG
Vim	CGTCCACACGCACCTACAG	GGGGGATGAGGAATAGAGGCT
Twist	CGGGTCATGGCTAACGTG	CAGCTTGCCATCTTGGAGTC
Zeb1	CATTTGATTGAGCACATGCG	AGCGGTGATTCATGTGTTGAG

Metabolomics details

Label-free targeted metabolomics

Agilent 1290 UHPLC and 6490 Triple Quadrupole (QqQ) Mass Spectrometer (LC-MS) was used in this study. Agilent MassHunter Optimizer and Workstation Software LC/MS Data Acquisition for 6400 Series Triple Quadrupole B.08.00 was used for standard optimization and data acquisition. Agilent MassHunter Workstation Software Quantitative Analysis Version B.0700 for QqQ was used for data analysis.

For reversed-phase chromatography (RPC), a Waters Acquity UPLC BEH TSS C18 column (2.1 x 100mm, 1.7µm) column was used with mobile phase (A) consisting of 0.5 mM NH4F and 0.1% formic acid in water; mobile phase (B) consisting of 0.1% formic acid in acetonitrile. Gradient program: mobile phase (B) was held at 1% for 1.5 min, increased to 80% in 15 min, then to 99% in 17 min and held for 2 min before going to initial condition and held for 10 min.

For hydrophilic interaction chromatography (HILIC), a Waters Acquity UPLC BEH amide column (2.1 x 100mm, 1.7µm) column was used with mobile phase (A) consisting of 20mM ammonium acetate, pH 9.6 in water; mobile phase (B) consisting of acetonitrile. Gradient program: mobile phase (B) was held at 85% for 1 min, increased to 65% in 12 min, then to 40% in 15 min and held for 5 min before going to initial condition and held for 10 min.

Both columns were at 40℃ and 3 µl of each sample was injected into the LC-MS with a flow rate of 0.2 ml/min. Calibration of TOF MS was achieved through Agilent ESI-Low Concentration Tuning Mix. Optimization was performed on the 6490 QqQ in the positive or negative mode for the RPC or HILIC respectively for each of 220 standard compounds to get the best fragment ion and other MS parameters for each standard. Retention time for each of 220 standards was measured from a pure standard solution or a mix standard solution. The LC-MS/MS method was created with dynamic dMRMs with RTs, RT windows and MRMs of all 220 standard compounds.

Key parameters of AJS ESI in both the positive and the negative acquisition modes are: Gas temp 275 ℃, Gas Flow 14 l/min, Nebulizer at 20 psi, SheathGasHeater 250℃, SheathGasFlow 11 l/min, and Capillary 3000 V. For MS: Delta EMV 200V or 350V for the positive or negative acquisition mode respectively and Cycle Time 500ms and Cell Acc 4V for both modes.

Metabolomics data post-processing and bioinformatic analysis

The QqQ data pre-processed with Agilent MassHunter Workstation Software Quantitative Analysis were post-processed for further quality control in the programming language R. We calculated coefficient of variation (CV) across replicate samples for each metabolite given a cut-off value of peak areas in both the positive and the negative modes. We then compared distributions of CVs for the whole dataset for a set of peak area cut-off values of 0, 1000, 5000, 10000, 15000, 20000, 25000 and 30000 in each mode. A noise cut-off value of peak areas in each mode was chosen by manual inspection of the CV distributions: 5000 for the positive mode and 0 for the negative mode. Each sample is then normalized by the total intensity of all metabolites to reflect the same protein content as a normalization factor. We then retained only those metabolites with at least 2 replicate measurements. The remaining missing value in each condition for each metabolite was filled with the mean value of the other replicate measurements. Finally, each metabolite abundance level in each sample was divided by the median of all abundance levels across all samples for proper comparisons, statistical analyses, and visualizations among metabolites. The statistical significance test was done by a two-tailed t-test with a significance threshold level of 0.05. The p-values were not adjusted in favor of more flexible biological interpretation. All other bioinformatics analyses including graphs and plots were also done using R/Bioconductor.

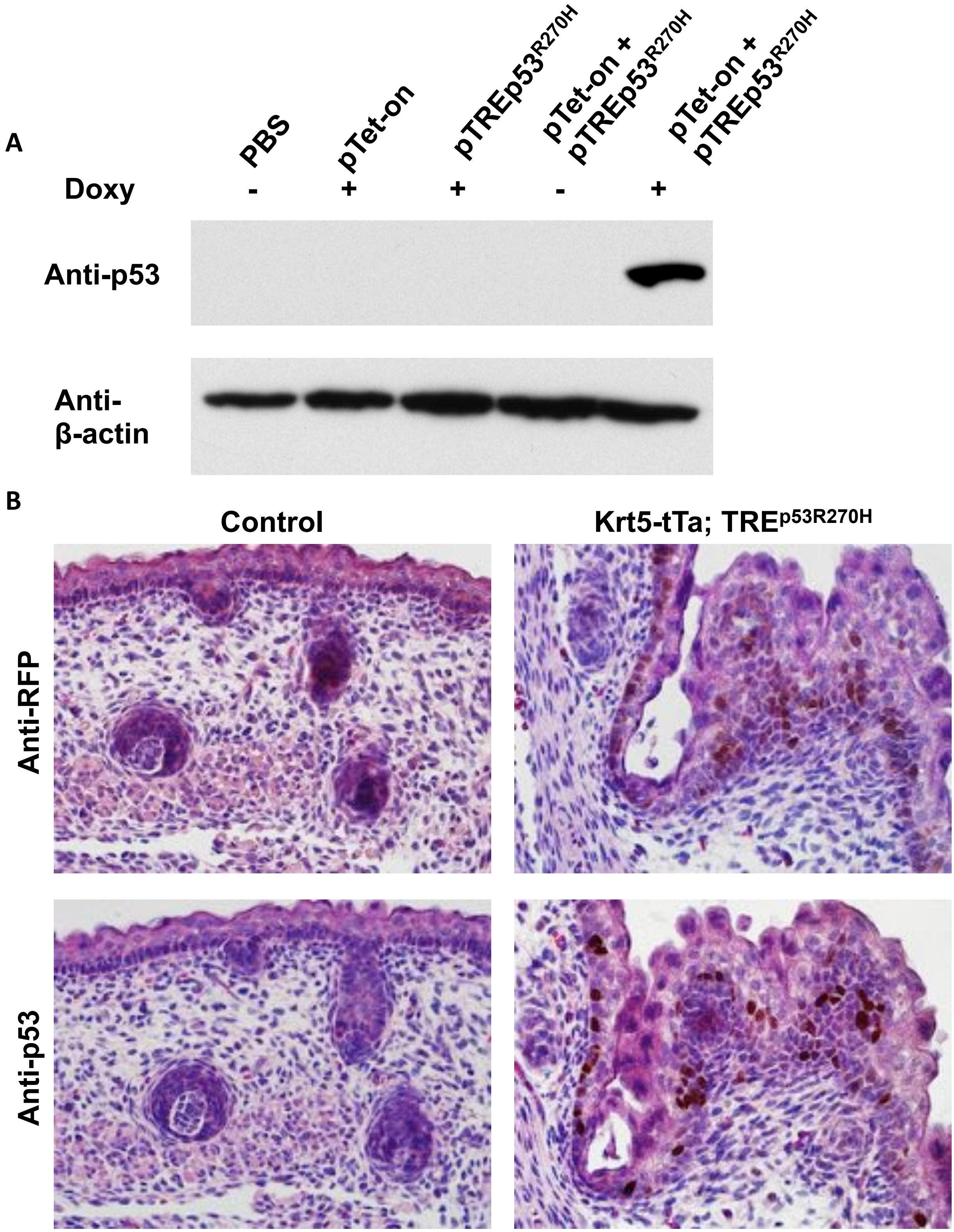

Supplemental Figure 1 – TREp53^{R270H} Validation *in vitro* and *in vivo*
(A) Western Blot for p53 to confirm p53^{R270H} expression upon dox administration. (B) IHC for RFP (dsRed) and p53 in Control and Krt5-tTa; TRE^{p53R270H} animal epithelium to confirm p53^{R270H} expression.

Mouse ID	Survival (Days)	Metastasis - Liver	Metastasis - Lung
A4924	**681**	**Y**	**Y**
A5251	**153**	**N**	**N/A**
A5567	**88**	**N/A**	**N/A**
A5777	**671**	**N/A**	**N/A**
A6378	**234**	**N**	**N/A**
A6397	**453**	**N**	**N**
A6467	**211**	**Y**	**N**
A6497	**195**	**N/A**	**N/A**
A7202	**362**	**N/A**	**N/A**
A7204	**275**	**Y**	**N**
A7206	**363**	**N/A**	**N/A**
A7208	**201**	**Y**	**N/A**
A7307	**273**	**N/A**	**N/A**
A7562	**574**	**Y**	**N**
A7573	**343**	**N/A**	**N/A**
A7577	**357**	**N/A**	**N/A**
A7582	**448**	**N**	**N**
A7693	**289**	**Y**	**N**
A7805	**502**	**Y**	**Y**
A8138	**140**	**N/A**	**N/A**
A8291	**355**	**N**	**Y**
A8570	**636**	**N/A**	**N/A**
A8800	**570**	**N/A**	**N/A**
A8807	**221**	**N/A**	**N/A**
A9182	**40**	**N/A**	**N/A**
A9521	**41**	**N/A**	**N/A**
A9966	**467**	**N/A**	**N/A**
A10118	**304**	**N**	**N**
A10157	**158**	**N/A**	**N/A**
A10196	**58**	**N**	**N**
A10197	**260**	**Y**	**Y**
A10363	**39**	**N/A**	**N/A**
A10407	**90**	**N/A**	**N/A**
A10410	**420**	**N/A**	**N/A**
A10589	**35**	**N/A**	**N/A**
A11053	**57**	**N/A**	**N/A**
A11306	**79**	**N/A**	**N/A**
A11384	**301**	**N**	**N**
A11430	**290**	**N/A**	**N/A**
A11431	**144**	**N**	**N**
A11586	**268**	**N**	**N**
A11662	**265**	**N/A**	**N/A**
A11746	**251**	**N/A**	**N/A**
A11798	**242**	**N/A**	**N/A**
A11813	**240**	**N**	**Y**
A10408	**274**	**N**	**N**
A12510	**42**	**Y**	**Y**
A12532	**168**	**N/A**	**N/A**
A12968	**45**	**N/A**	**N/A**
A13076	**59**	**N/A**	**N/A**
A12988	**83**	**N/A**	**N/A**
A13721	**247**	**N**	**N**
A14649	**92**	**N/A**	**N/A**
A15564	**61**	**N/A**	**N/A**

Supplemental Figure 2 – Details of KCip53 mouse survival
Table listing survival (in days) of KCip53 animals and evaluation of metastasic disease. N/A indicates that no histopathologycal analysis was possible. N=54 KCip53 animals.

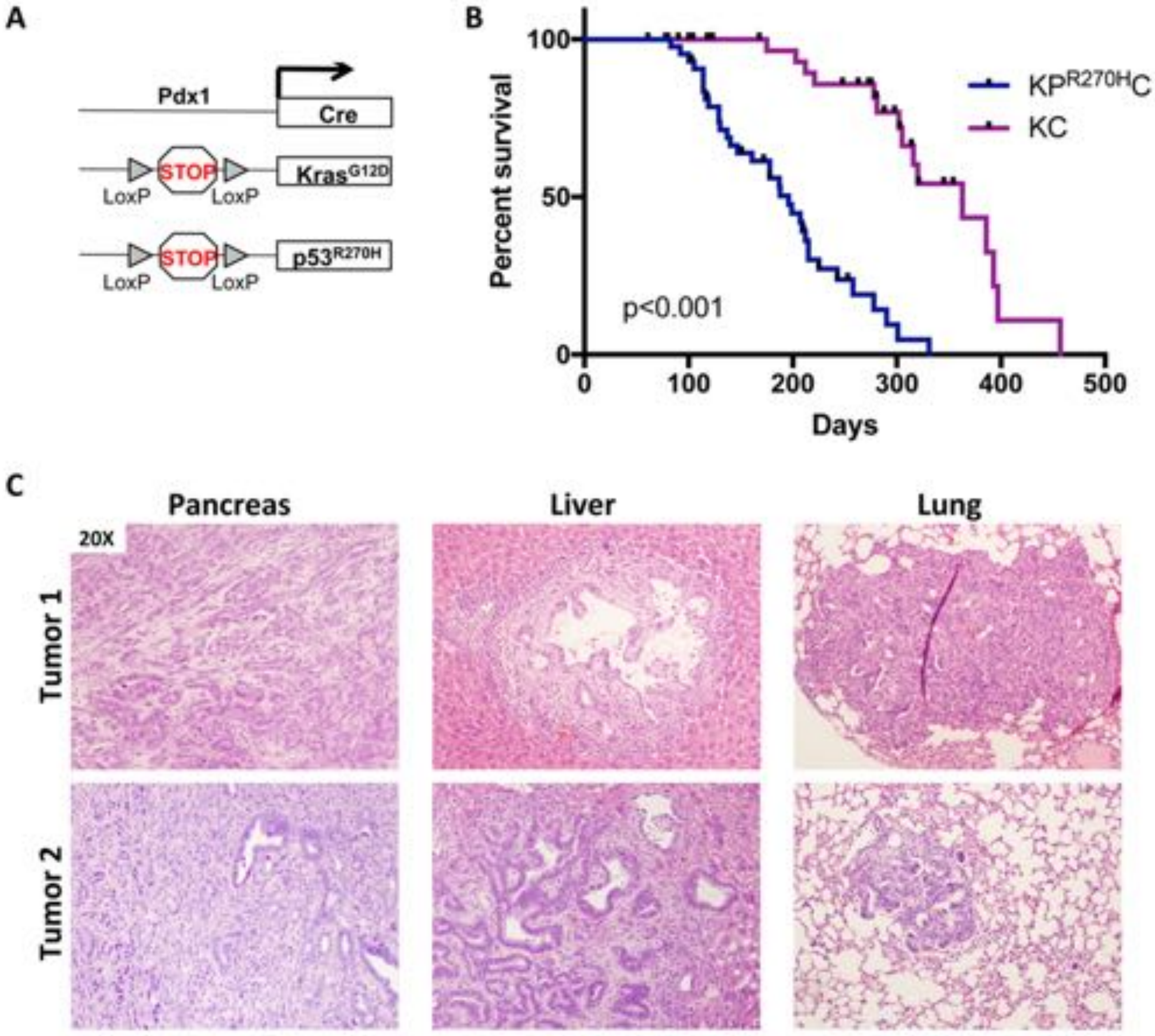

Supplemental Figure 3 – KP^{R270H}C animals develop metastatic pancreatic tumors

(A) Scheme of Pdx1-Cre; LSLKras^{G12D}; Tp53$^{R270H/+}$ animals, termed KP^{R270H}C here (B) Survival curve for KC and KP^{R270H}C animals. (C) H&E from pancreas, liver and lung from two separate KP^{R270H}C animals with tumors, shown at 20x magnification. N=43 KP^{R270H}C and 39 KC. Survival significance analysis by Log Rank Test.

A

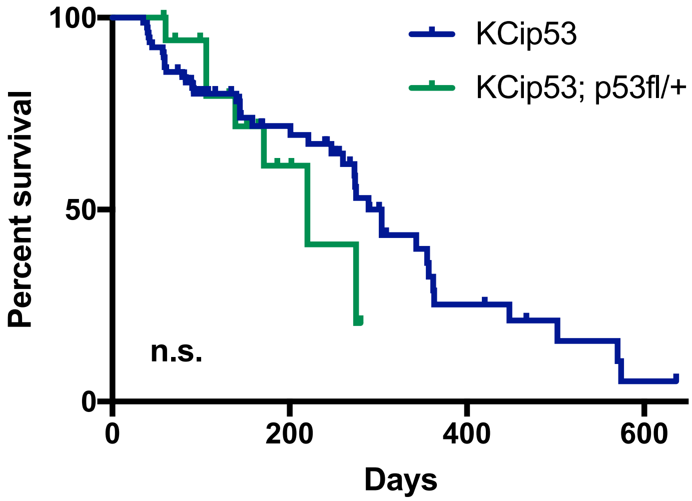

B

Mouse ID	Age (Days)	Tumor	Mets?
A13458	275	Y	N
A13720	220	Y	N
A13984	106	Y	N
A14561	171	No Histology	
A14857	106	No Histology	
A14915	139	Y	N
A15822	60	No Histology	

C

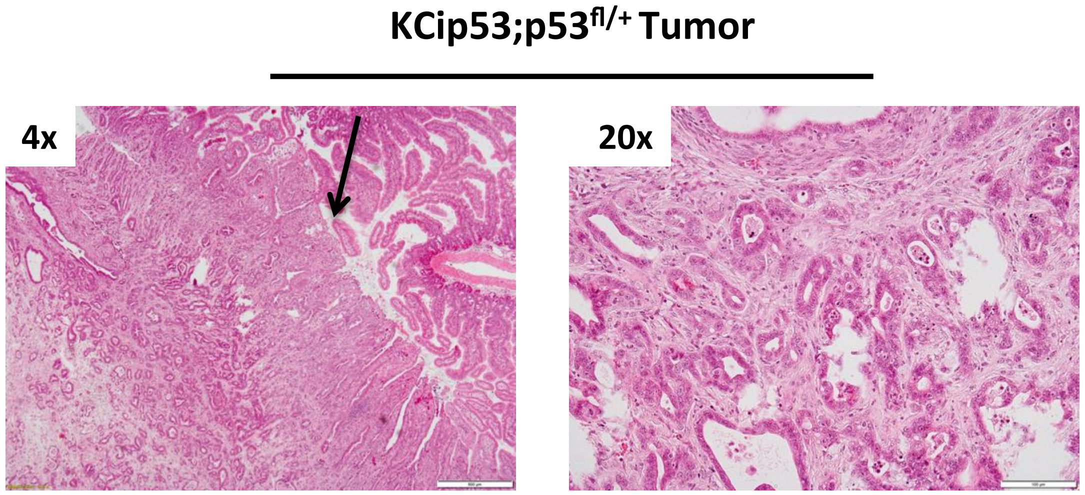

Supplemental Figure 4 – KCip53;p53$^{fl/+}$ animals develop locally invasive pancreatic tumors

(A) Survival of Ptf1aCre; LSL-Kras^{G12D}; TREp53^{R270H}; R26$^{rtTa/rtTa}$; p53$^{flox/+}$ animals, termed KCip53; p53$^{fl/+}$ here. (B) Table detailing survival length of KCip53; p53$^{fl/+}$ animals, with metastasis information for animals from which histology was available. (C) H&E from the tumor of a KCip53;p53$^{fl/+}$ animal. Arrow indicates area of local invasion into the adjacent intestine. Histology shown at both low (4X) and high (20x) magnification, as noted on images. n= 18 KCip53; p53$^{fl/+}$ animals in survival curve, 7 animals with tumors. Survival significance analysis by Log Rank Test.

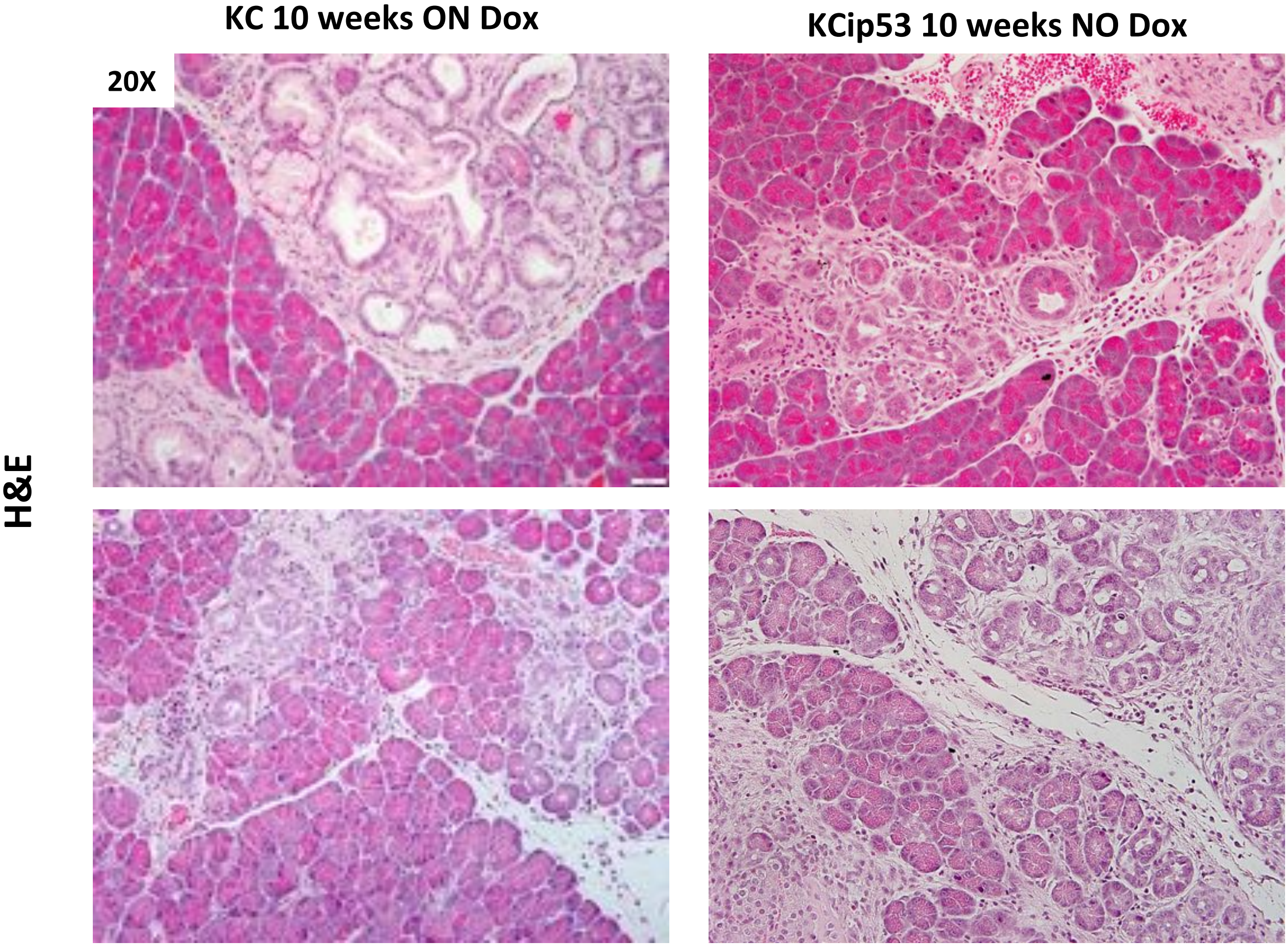

Supplemental Figure 5 – KCip53 histology at ten weeks of age without dox
H&E analysis of KCip53 animals never on dox at 10 weeks of age compared to KC animals on dox at 10 weeks of age. Histology images shown at 20x magnification. n=3 KC on dox, 4 KCip53 never on dox.

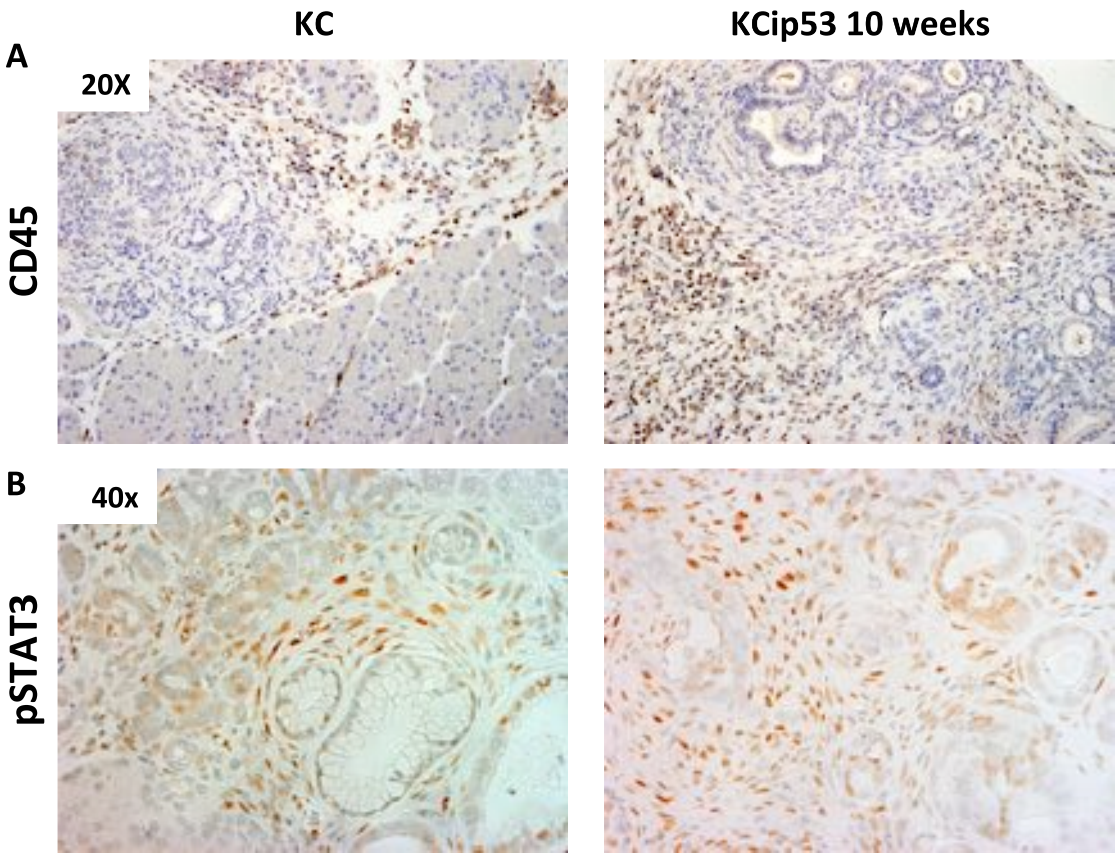

Supplemental Figure 6 – KCip53 histology at ten weeks of age
IHC for (A) CD45 (20x magnification) and (B) pSTAT3 (40x magnification) in 10-week-old KC or KCip53 animals on dox. N=3-6 animals per condition. For CD45 at least 5 high power fields were analyzed per animal.

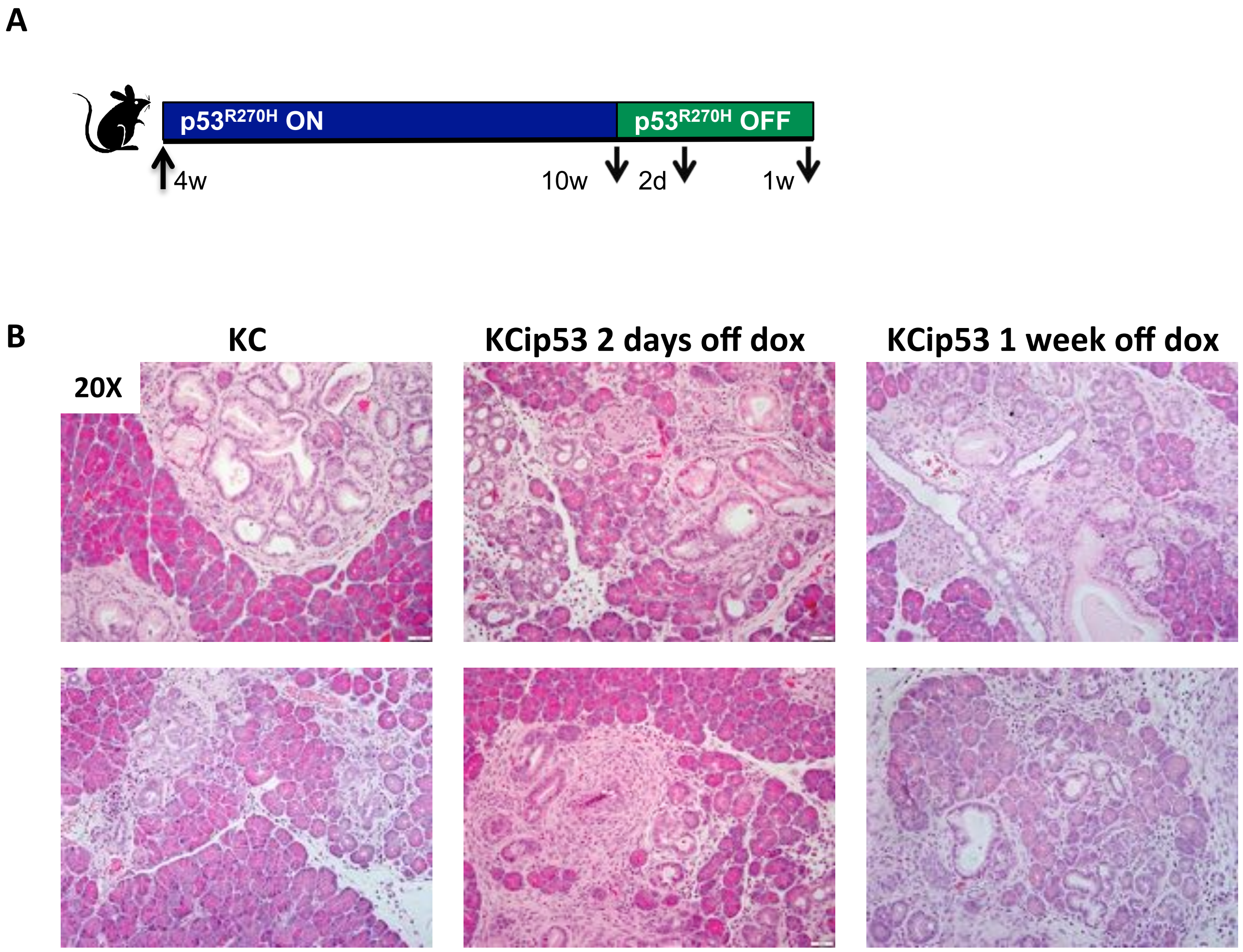

Supplemental Figure 7 – KCip53 histology at two days and one week off dox
(A) Scheme for dox treatment in KCip53 animals. (B) H&E analysis of KC and KCip53 animals taken off dox. Histology images shown at 20x magnification. n=3-6 animals per condition.

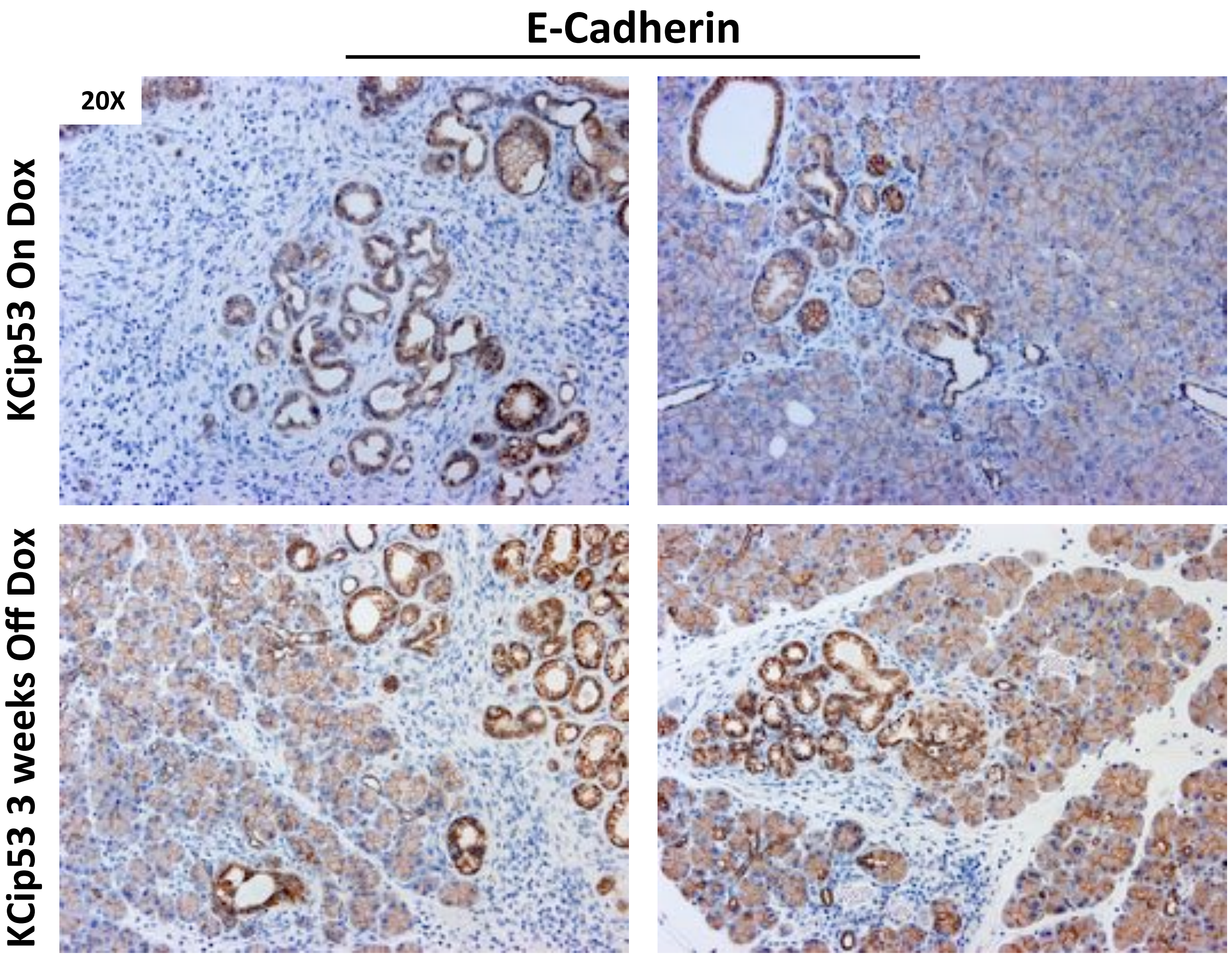

Supplemental Figure 8 – E-Cadherin is upregulated in KCip53 animals removed from dox
IHC for E-Cadherin in KCip53 animals on dox and KCip53 animals 3 weeks after removal from dox, shown at 20x magnification. n=3-6 animals per condition.

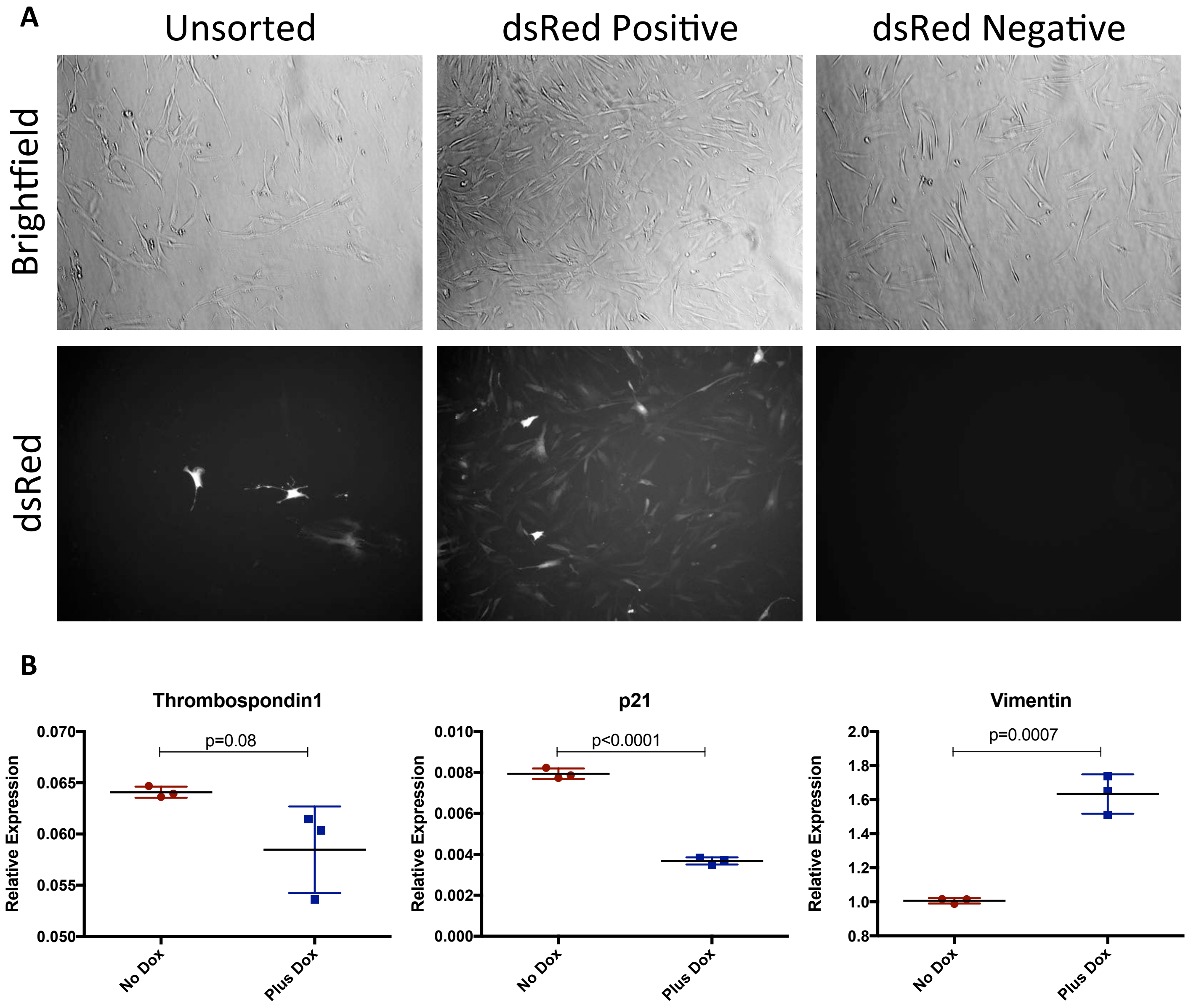

Supplemental Figure 9 – KCip53-1 Cell line validation by dsRed and p53 gene target expression

(A) Bright field and dsRed fluorescence images of KCip53-1 cells before and after cell sorting for dsRed expression. (B) qRT-PCR analysis for p53 targets in KCip53-1 cells grown with or without dox. For all qPCR unpaired t-test with Welsh's correction was used, and data is represented as mean with SD. N=3.

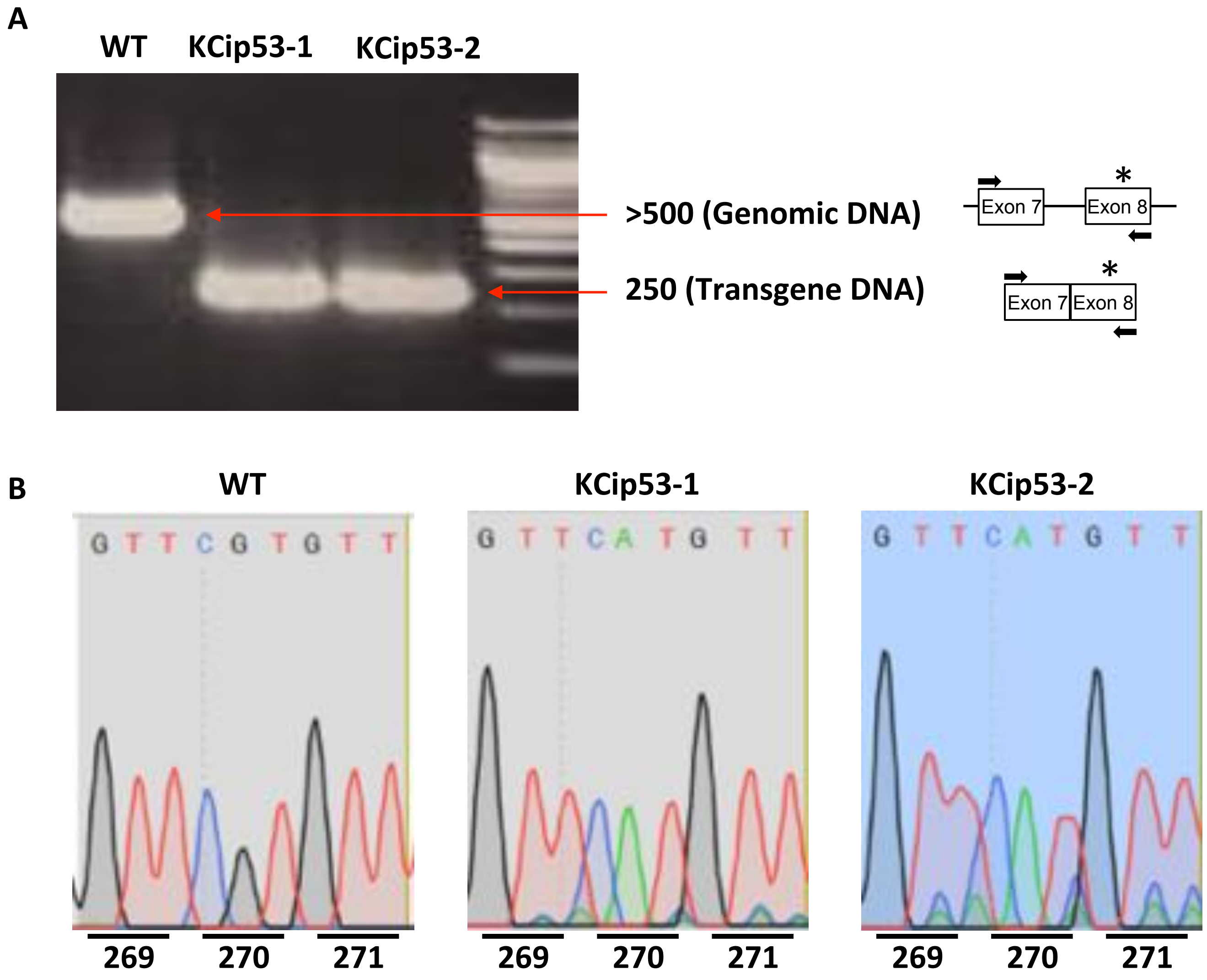

Supplemental Figure 10 – KCip53 Cell line sequencing of *Trp53* exon 7-8. (A) PCR amplification of p53 amino acid 270 region in mouse pancreatic cancer cells that are wild type at that region compared to KCip53 cell lines, and scheme of primer placement. (B) Sequencing results from same cell lines showing the wild type sequence at codon 270 (*left*) and a single sequence containing the point mutation at codon 270 in both KCip53 cell lines *(middle and right).*

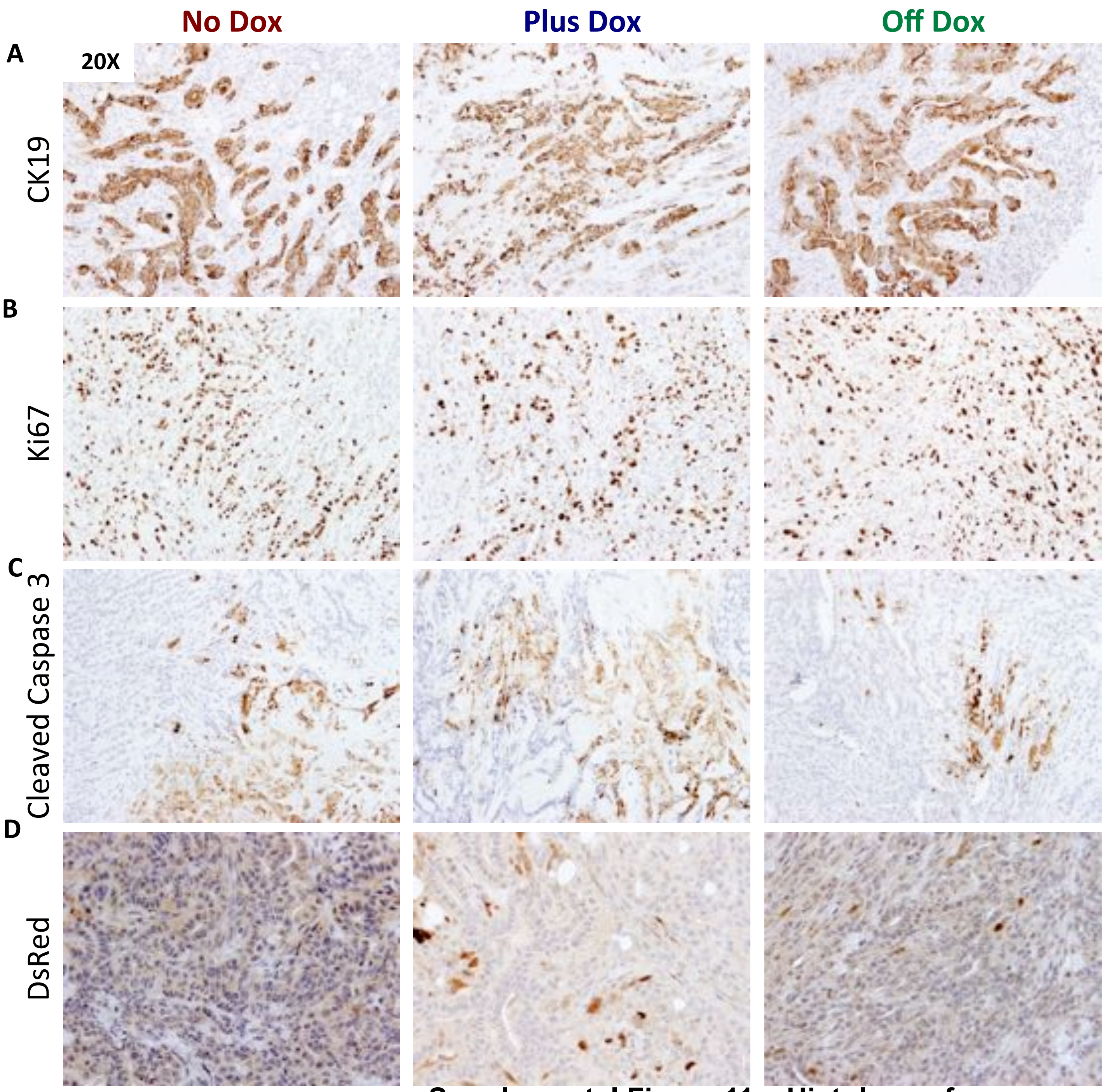

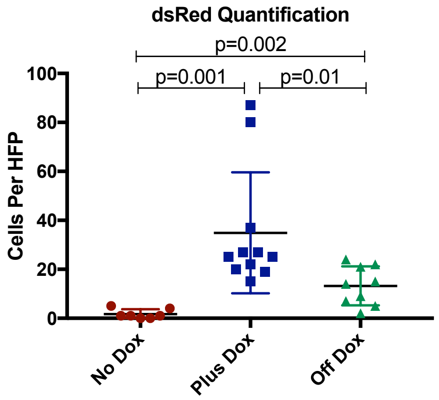

Supplemental Figure 11 – Histology of subcutaneous tumors from KCip53-1 cells
IHC analysis of subcutaneous tumors from KCip53-1 cells including (A) CK19, (B) Ki67, (C) Cleaved Caspase 3 and (D) dsRed. Quantification of number of dsRed expressing cells per high power field in final subcutaneous tumors from KCip53-1 cells, analyzed using multiple unpaired t-tests. Represented as mean with SD. All histology images shown at 20x magnification. n=6-8 tumors per condition, experiment repeated three times.

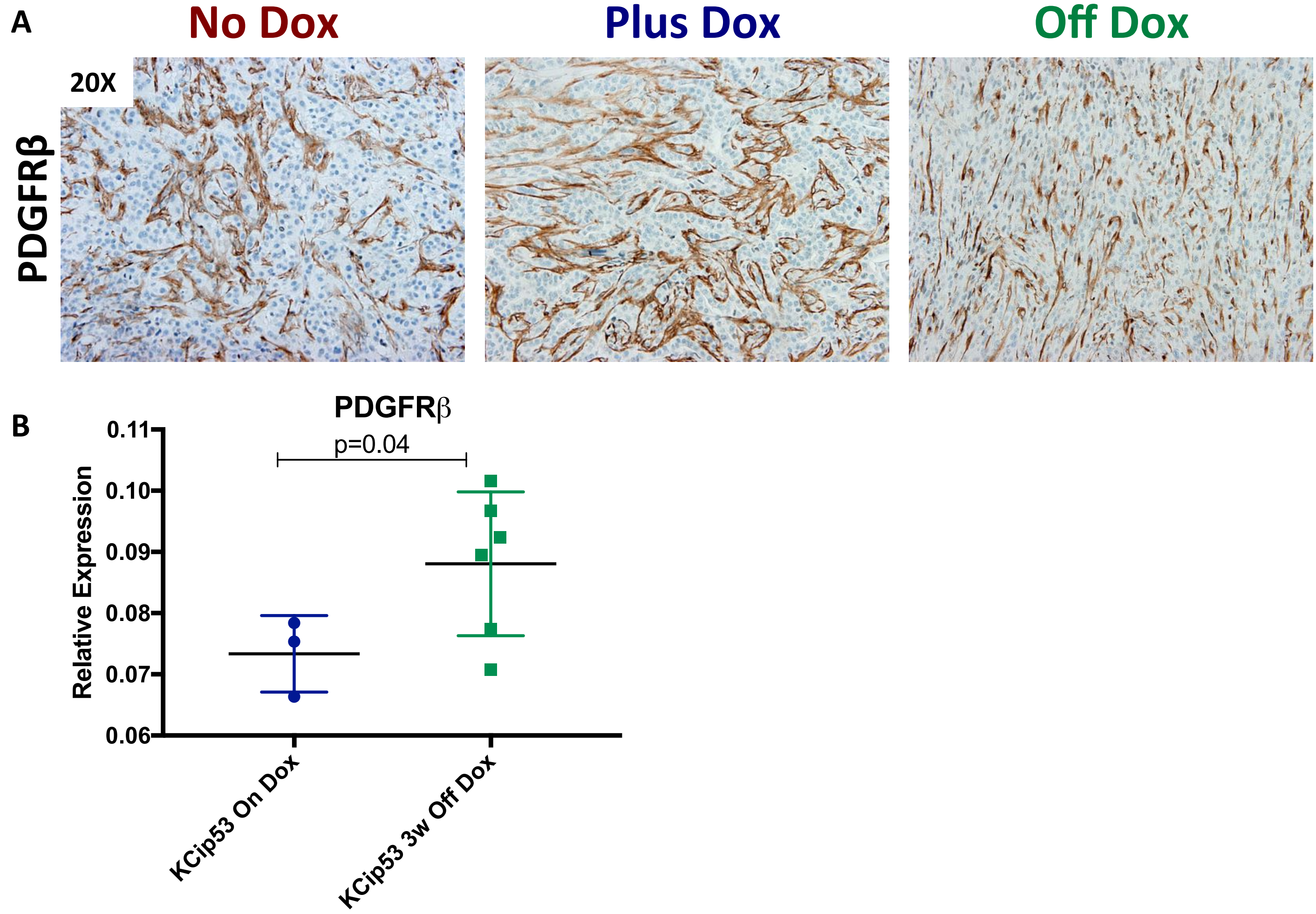

Supplemental Figure 12 –PDGFRb expression in KCip53 tumors (A) IHC analysis for PDGFRβ (20x magnification) of final subcutaneous tumors grown from KCip53-2 cells shows prevalent expression in the stromal compartment. N=6-8 tumors per group. (B) qRT-PCR analysis of PDGFRβ levels in KCip53 animals on dox compared to those removed from dox for 3 weeks. A trend towards higher expression upon dox removal might be explained by a change in the relative stroma composition. Unpaired t-test with Welsh's correction was used, and data is represented as mean with SD. N=3-6 animals per condition.

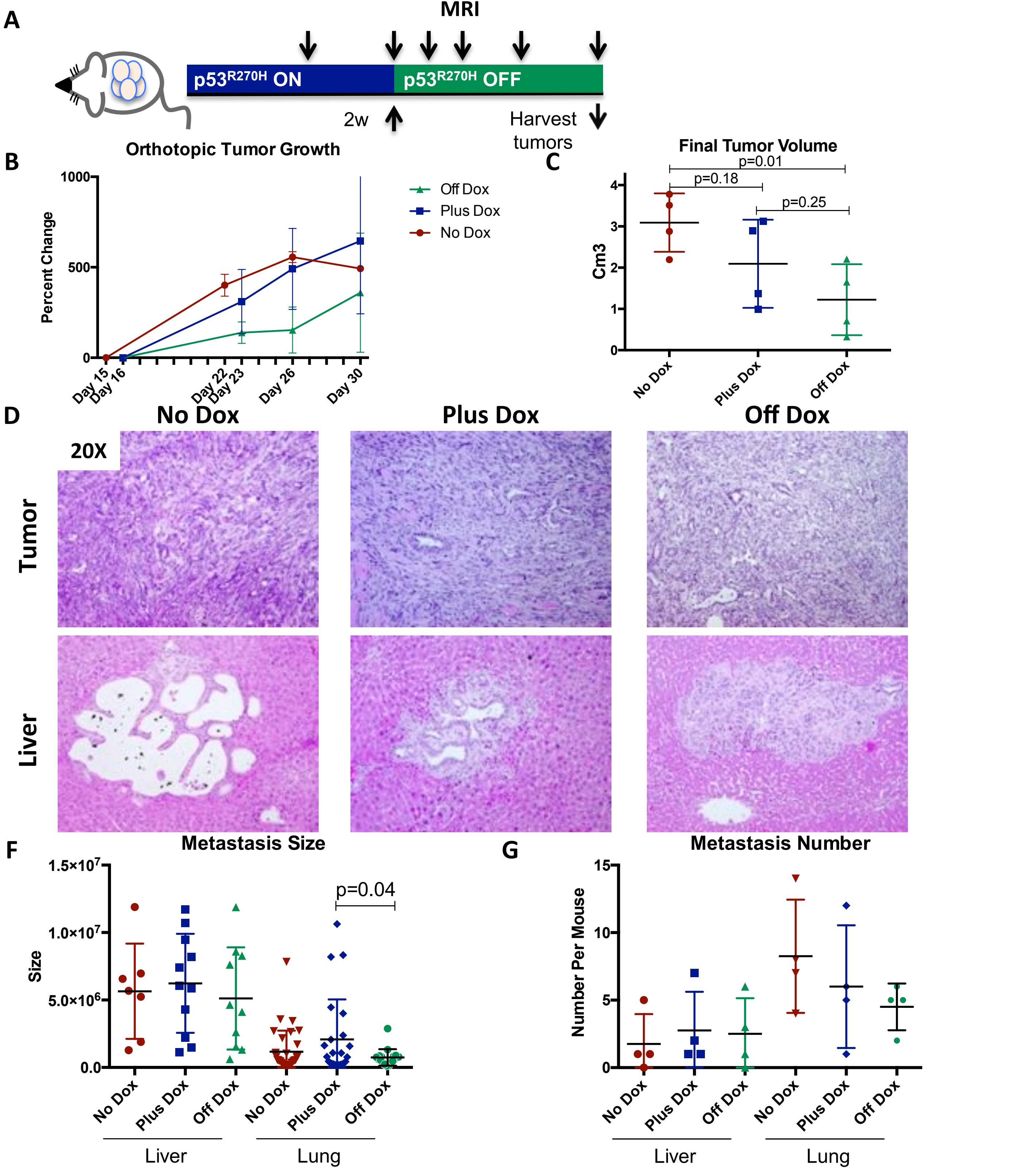

Supplemental Figure 13 - Assessing the effect of mutant $p53^{R270H}$ expression on orthotopic tumor growth

(A) Scheme for orthotopic tumor growth and MRI measurement (C) Final tumor volume. (D) H&E for final orthotopic tumors and representative liver metastases (20x magnification). (E) Quantification of average metastasis size. (F) Quantification of average number of metastases. N=3-4 animals per group. Analyzed by multiple unpaired t-tests, shown as mean with SD.

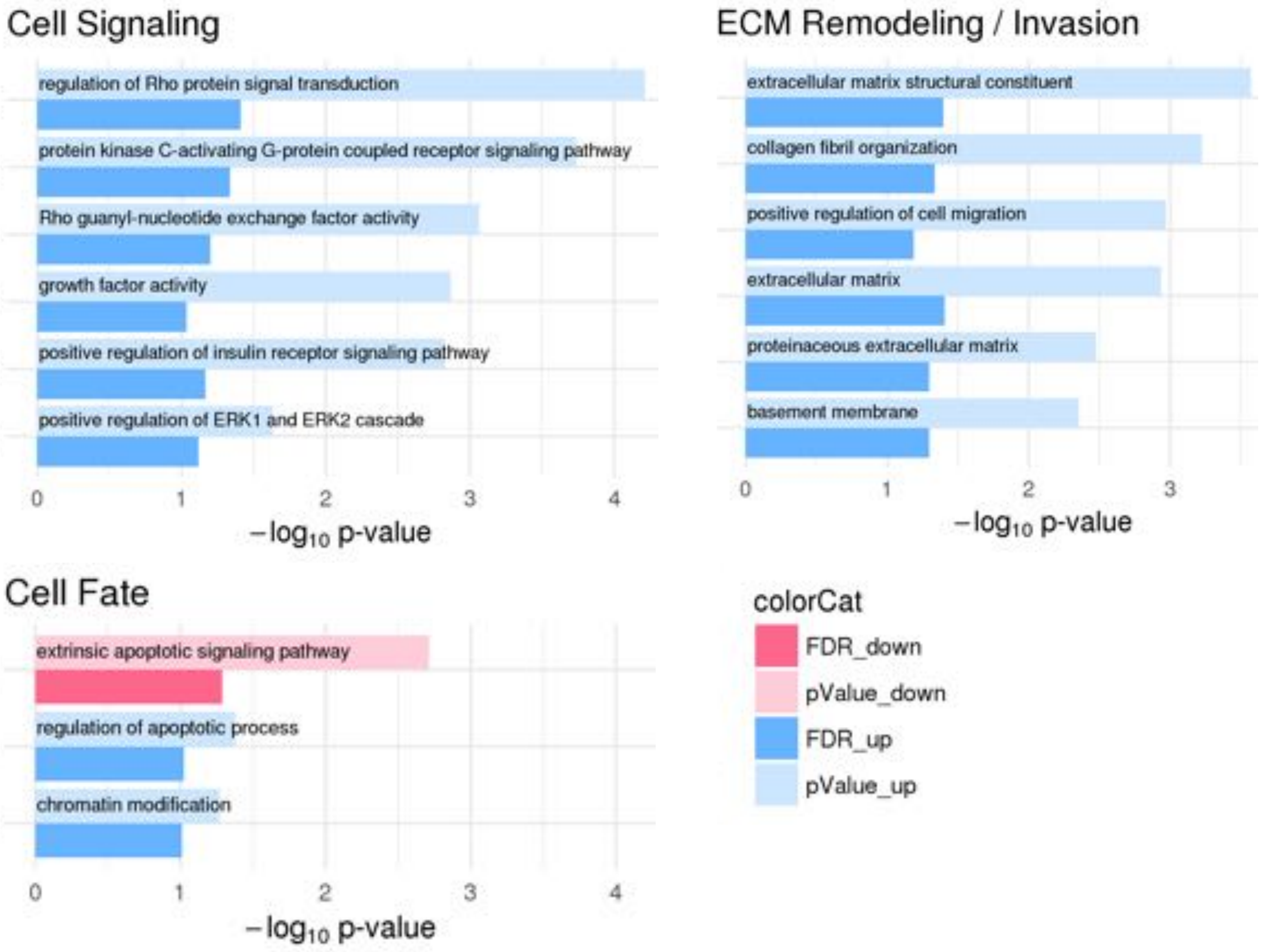

Supplemental Figure 14 – Further Differentially Regulated Pathways in Plus Dox Tumors
Pathway analysis in “No dox” compared to “Plus dox” tumors. N=4 samples per group for RNA sequencing.

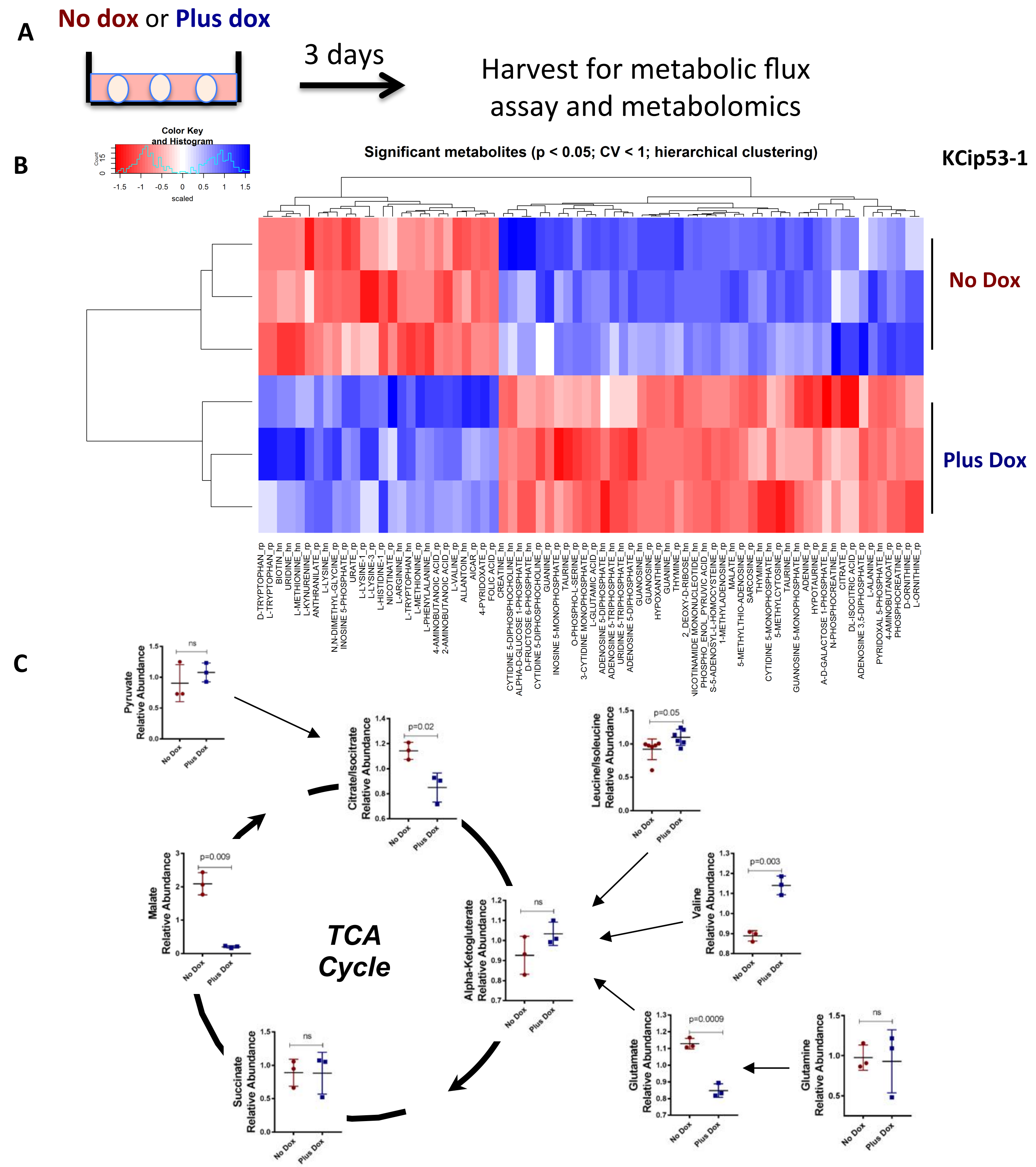

Supplemental Figure 15 – Metabolomics Analysis of KCip53 cells

(A) Scheme for cell growth for metabolomics analysis (B) Heatmap of the significant metabolites changed in KCip53-1 cells with p53^{R270H} (Plus dox) or without (No dox). (C) Summary of changed TCA intermediates and substrates. Unpaired t-test with Welsh's correction was used, and data is represented as mean with SD. N=3 samples per group.

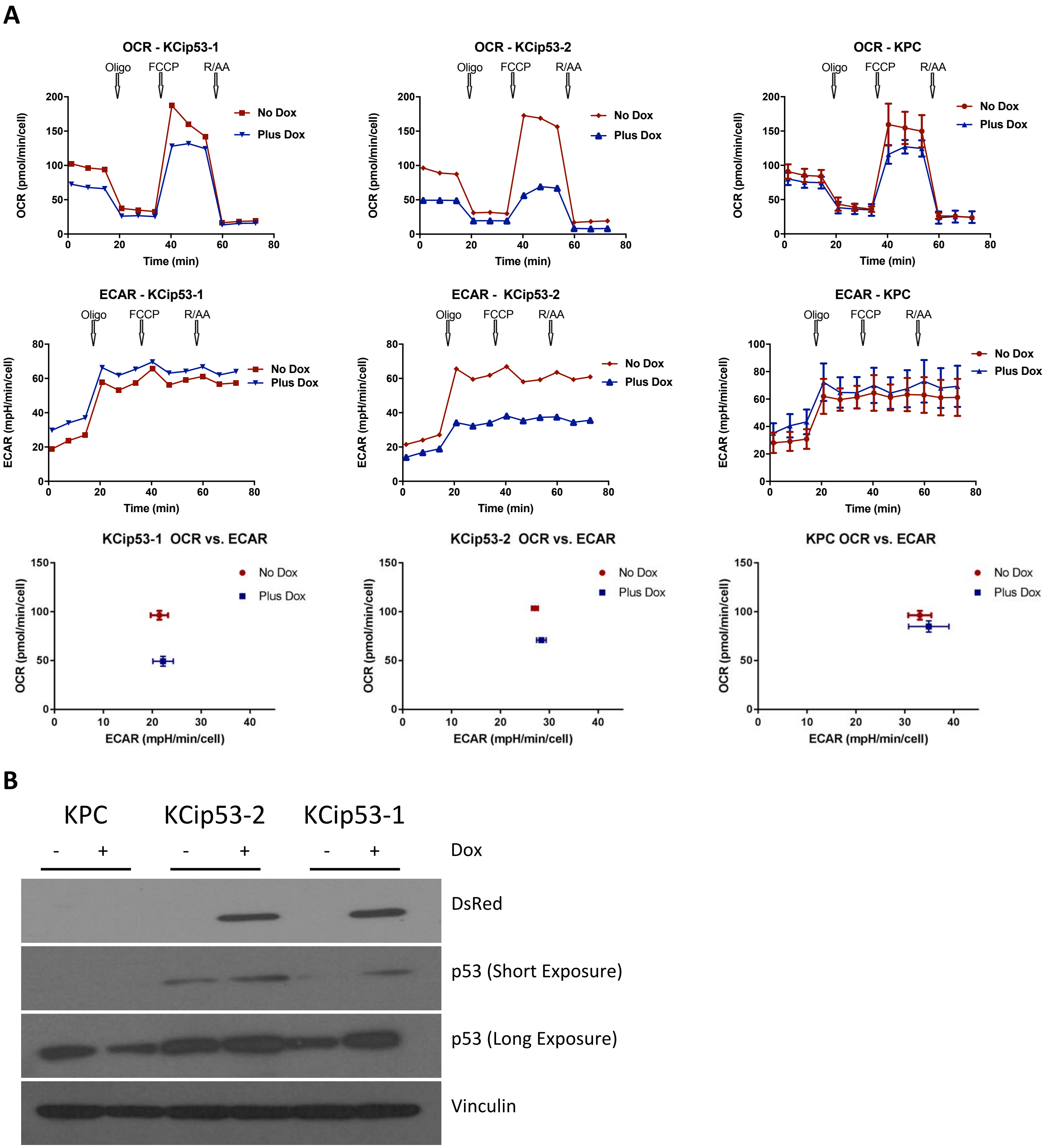

Supplemental Figure 16 – Changes in OCR but not ECAR in KCip53 cell lines with p53^{R270H} expression

(A) Traces showing change in OCR and ECAR during a mitochondrial stress test in KCip53-1, KCip53-2, and KPC cells grown with or without dox. N=5 samples per condition. (B) Western blot analysis of KPC, KCip53-1, and KCip53-2 cell lines for dsRed expression and p53 expression

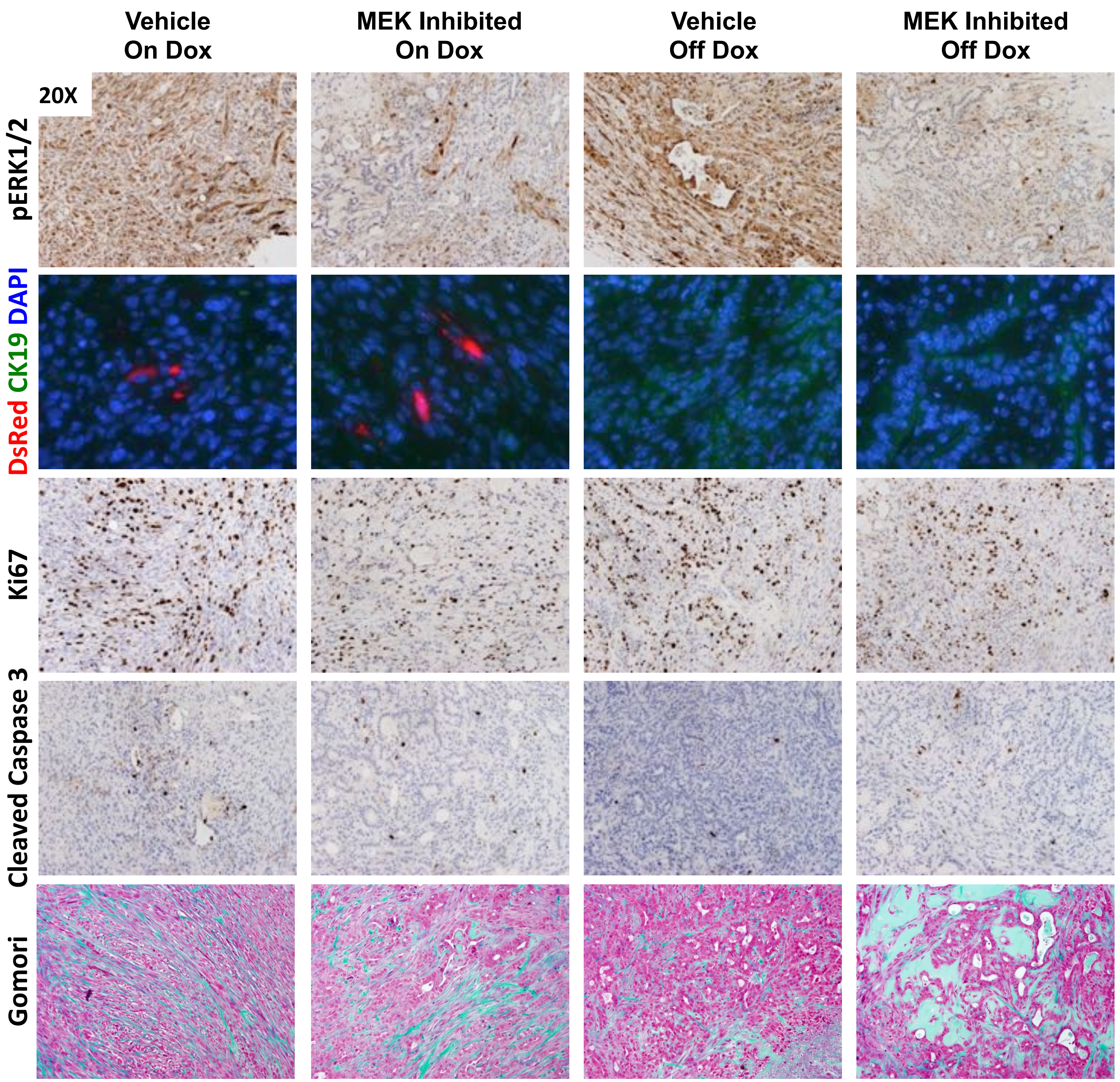

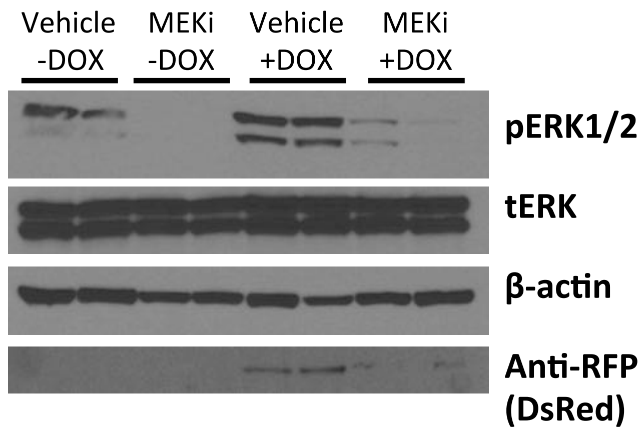

Supplemental Figure 17 – Histology of KCip53-1 subcutaneous tumors with MEK inhibition and $p53^{R270H}$ expression
Resulting histology from final tumors from MEK inhibition and on and off dox subcutaneous tumor growth groups (20x). N=10 tumors per group. IHC for pERK1/2, Ki67 and Cleaved Caspase 3. IF for dsRed as a surrogate for $p53^{R270H}$ expression. Western Blot for pERK1/2 to confirm MEK inhibition and dsRed to confirm $p53^{R270H}$ expression. Each lane represents lysate from one subcutaneous tumor.

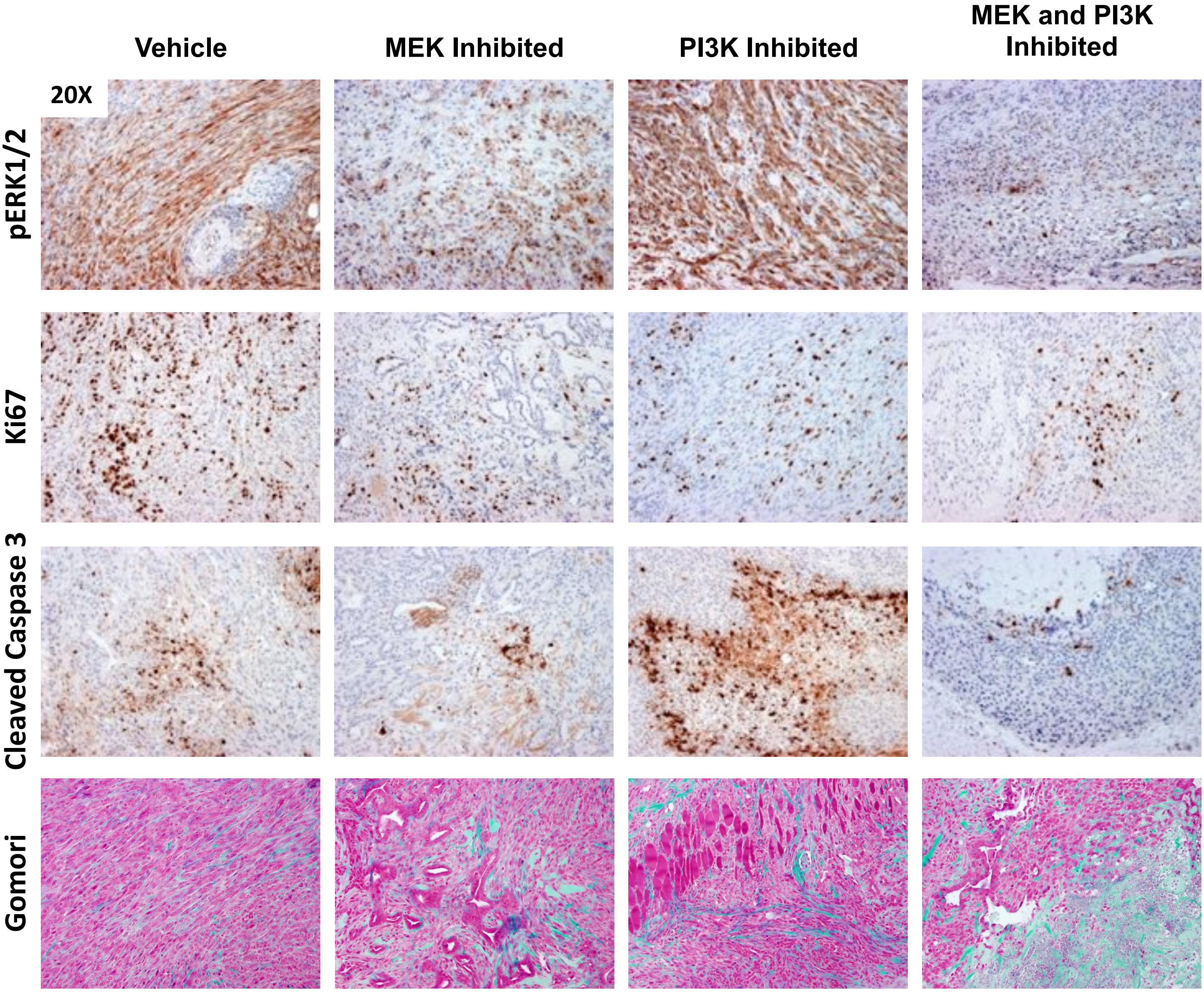

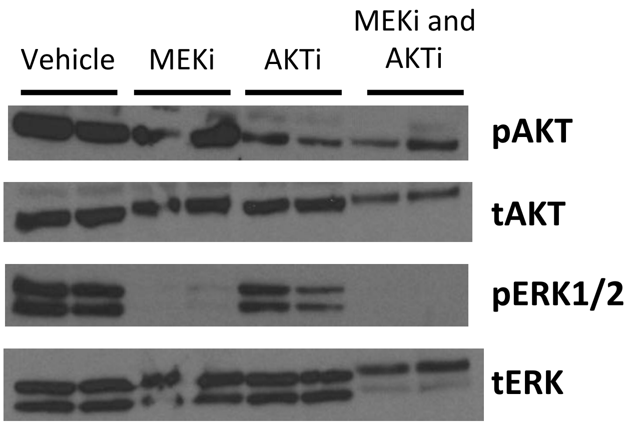

Supplemental Figure 18 – Histology of KCip53-1 subcutaneous tumors with MEK and/or PI3K inhibition
Resulting histology from final tumors from MEK and/or PI3K inhibition in subcutaneous tumor growth groups, shown at 20x magnification. N=8 tumors per group. IHC for pERK1/2, Ki67 and Cleaved Caspase 3. Western Blot for pAKT and total AKT to confirm PI3K inhibition and pERK1/2 to confirm MEK inhibition. Each lane represents lysate from one subcutaneous tumor.
